# Supplementary material for: Device-based measurement of physical activity and sedentary behaviour after critical illness: A scoping review
Source: PLoS One. 2025 Jun 3;20(6):e0322339. doi: 10.1371/journal.pone.0322339 (PMC12133016; doi:10.1371/journal.pone.0322339)
Supplement: S2 Table — (DOCX) [file pone.0322339.s002.docx]

| **Table S2 Study characteristics** | | | | | | |  |
| --- | --- | --- | --- | --- | --- | --- | --- |
|  | ***Authors, Year*, Country *Study: Title/type*** | ***Setting*** | ***Population, Main ICU diagnosis/reason for admission, Number (n=), M:F, Age, BMI (kg/m*²*), Ethnicity, Duration of mechanical ventilation*** | ***Inclusion/exclusion criteria*** | ***Parameters used to measure physical activity and or sedentary behaviour*** | ***Control Group*** |  |
| 1 | Baldwin *et al*., 2020, Australia   The sedentary behaviour and physical activity patterns of survivors of critical illness over their acute hospitalisation: An observational study  Observational cohort study | ICU, followed by acute care | Reason for ICU: Medical (n = 28 / 70%) / surgical (n = 12 / 30%);  N=40 M = 24 (60%) / F = 16 (40%);  BMI 29.2(6.5) kg/m²;  95% Caucasian / 5% ATSI  Median duration of mechanical ventilation = 11 [IQR 8-21] days | Required MV ≥5 days, ≥ 18 years of age,  excluded if they were unable to understand or comprehend English, unable to ambulate independently ≥10 metres in past three months, receiving palliative care. | Time in activity | N/A |  |
| 2 | Gandotra *et al.,* 2021, USA   Activity in survivors of the Intensive care unit.  Prospective observational study | ICU, ward setting and in community (7 days after hospital discharge) | Reason for ICU: not stated.   Duration mechanical ventilation: up to 7 days or less via ETT or NIV mask.  Enrolled at time of undergoing spontaneous breathing trials or <24hr after extubation.   n=22. Of n=15 analysed: Age: 68+9.6, 47% (n=7) female.   BMI 30.7+6, kg/m²;  APACHE III median score 76 [IQR 46-83].  Median duration (days) mechanical ventilation 2 [IQR 1-3].   Ethnicity: not stated | Previously independent adults, aged 55 or older, MV for 7 days or less. Enrolled 24 hours or less after extubation.  Exclusion criteria: Pregnancy, inability to walk without assistance prior to acute illness, prehospitalisation cognitive impairment/non verbal, history of stroke, BMI above 50kg/m², body mass less than 60kg, Neuromuscular disease that could impair weaning, liver disease or a fracture limiting mobility. Also hospitalisation over 14 days, receiving any type of renal therapy or cancer therapy in the previous 6 months. | Step counts | N/A - All received standard care for mobility. |  |
| 3 | Mc Nelly *et al.,* 2016, UK  An exploratory study of long-term outcome measures in critical illness survivors: Construct validity of physical activity, frailty and health related quality of life measures.  Prospective outcome study | At home, 18 months post ICU discharge | Admission diagnosis sepsis 13(48.1%), trauma 6(22.2%), intracranial bleeding 2(7.4%) cardiogenic shock 6(22.2%)  N=27. M 13(48.1),  Age 54 (46.6-61.6),  BMI NR, Ethnicity NR,  duration of mechanical ventilation 7 days (2-24). | inclusion: invasively ventilated for more than 48 hours and on the ICU >7 days. | Step counts | Norm based values (for HRQoL questionnaire), healthy controlled scores (for daily step counts accelerometery), controls for frailty (clinical frailty score) were non-morbid, age and gender matched to survivors. |  |
| 4 | Baldwn *et al.,* 2018, Australia  Physical activity of ICU survivors during acute admission: Agreement of the ActivPAL with observation.  Prospective observational study | ICU discharge | N=8, M 2: F 6;  Median age 56 (IQR 1-3 48-65)  BMI not stated;  Duration of MV not stated,  reason for admission not stated. | Required MV ≥5 days, ≥ 18 years of age, able to transition from sitting to standing with a maximum assistance of 2, able to attempt standing/stepping,  Excluded if they were unable to provide informed consent, unable to ambulate independently ≥10 metres in past three months, receiving palliative care. | Step counts | N/A |  |
| 5 | Borges *et al*., 2015, Brazil  Physical activity, muscle strength, and exercise capacity 3 months after severe sepsis and septic shock.  Prospective cohort study | ICU to hospital discharge | N=72, M 36: F 36;  BMI, kg/m²; 25.5± 4.8;  Duration of MV (days) 7.5 ± 6.2;  reason for admission not stated. | Included 18 years or older, ability to walk without assistance before hospitalisation, referred to ICU with sepsis or shock within 24 hours of evolution in another unit, only patients who were able to perform at least two physical assessments at time of discharge.  Exclusion, previous or current history of stroke with physical limitations, neuromuscular disease, traumatic brain injury, spinal cord injury, multiple traumas with fractured limbs or limb amputation, or any other disease which could affect physical assessments. Also terminal patients or those with persistent coma, patients who did not attend the 3 month follow up were also excluded. | Step counts | Control group of 50 healthy sedentary subjects. The control group consisted of the same sexes, age (±6 years) and BMIs (±2 kg/m2) |  |
| 6 | Van Bakel *et al*., 2022, Netherlands  High levels of sedentary time in patients with COVID-19 after hospitalisation.  Prospective cohort study | Hospital discharge 3 to 6 months | N=37, M:29 (78%) F:8 (22%),  Age ≤ 62: 19 (51%), >62: 18 (49%);  BMI: ≤ 26.8, 18 (49%), >26.8, 19 (51%);  Ethnicity: NR; Admitted to ICU: 13 (35%),  MV: N 11 (30%), duration of MV 16 ±7 (days).  Main diagnosis: COVID-19 | Post-hospitalised patients with COVID-19, between April 1st - May 12th 2020. | Time in activity,  activity intensity | N/A |  |
| 7 | Munro *et al.,* 2021, USA  Sleep and activity patterns are altered during early critical illness in mechanically ventilated adults.  Observational cohort study | ICU ward | N=31; M: 13 (41.9%), F:18 (58.1%);  mean age: 59.6 (±17.3);  White/caucasian26 (83.9%), Black/African-American 5(16.1%); Hispanic/Latino 21 (67.7%), Non-Hispanic 10 (32.3%);  Reasons for admission; Medical: 13 (41.9%), Surgical: 9 (29%), Trauma: 6 (19.4%), Neurological: 1 (3.2), Cardiac 2 (6.5%);  ICU LOS mean 17 days;  Mean days MV: 12.5 | Inclusion criteria: MV adults 18 years or older; enrolment within 48 hours of intubation and ICU admission; must have informed consent provided in English or Spanish; a family member must speak English or Spanish.  Exclusion criteria: Dementia, anticipation (by clinician) of imminent death, medical contraindication, inability to speak English or Spanish. | Activity counts | N/A |  |
| 8 | Plekhanova *et al*., 2022, UK  Device-assessed sleep and physical activity in individuals recovering from a hospital admission for COVID-19: a multicentre study.  Longitudinal cohort study | Post hospital discharge, 8 months | N=715; M:462 (64.61%), F:253 (35.84%);  Mean age (years) men; 60, Women 58;  BMI kg/m², Men 30.4, women: 32.2;  ethnicity: White: 499 (69.7%), South African: 92 (12.86%), Black: 49 (68.53%) Other: 43 (6%);  151 (32.7%) men & 66 (26.1%) women received MV, duration not specified. | Inclusion criteria; Adult ≥ 18 years; discharged from NHS hospitals following a confirmed diagnosis of COVID-19,  Exclusion criteria: confirmed diagnosis of a pathogen un related to the study, attended A&E but were not admitted, had a life limiting illness with life expectancy less than 6 months. | Time in activity,  activity intensity | Office workers and individuals with type 2 diabetes. |  |
| 9 | Anderson *et al.,* 2019, UK  A study investigating the validity of an accelerometer in quantification of step count in adult hospital inpatients recovering from critical illness.  Prospective observational study | Ward following discharge from ICU | n=24, 4 declined participation.  M: 13 (65%), F:7 (35%);  Age 62.3+11.5 (IQR 39-82);  BMI: 25.9+6.1 (16.9-38.3).  Ethnicity NR;  Ventilation (days) 15.0 (5.50, 36);  ICU LOS (days) 21.0 (8.25, 42.75) | Inclusion: Adults>18yrs, required >48hrs mechanical ventilation, able to mobilise independently or assistance of 1 person or a walking aid, understand study information and provide written informed consent.  Exclusion: unwilling to wear accelerometer for 3 hours or if refused to be observed during this period. | Step counts | N/A |  |
| 10 | Camus-Molina *et al*., 2020, Chile   Construct validity of the Chilean-Spanish version of the functional status score for the intensive care unit: A prospective observational study using actigraphy in mechanically ventilated patients.  Prospective observational study | ICU | Reason for admission, sepsis 9 (30%), coma 6(20%), oncological 6(20), hypovolaemic shock 4(13.3) respiratory 3(10), transplant 1(3.3) trauma 1 (3.3)  n=30, Female 17(59),  age 64.5yr 25th-75th percentile 55-74,  BMI 25.7 25th - 75th percentile 23.7-27.1,  ethnicity NR,  duration of MV 2.6 days (1.6-5.2) | Inclusion: Adults>18yrs, mechanically ventilated.  Exclusion: limitation of life sustaining care, plegia in 1 or more limbs, invasive ventilation in another centre for more than 24 hours, limb amputation, patient readmitted to ICU who was already enrolled in the study, patient awakening in the first 24 hours since ICU admission (to ensure at least 24h of actigraphy since ICU admission and awakening), previous functional dependence (FSS-ICU score<30), able to consent after ability to follow 5 simple commands and being delirium free) | Activity counts,  time in activity | No |  |
| 11 | Grap *et al.,* 2005, USA  Actigraphy- in the critically ill: Correlation with activity, agitation, and sedation.  Prospective descriptive correlational study. | Medical respiratory ICU and coronary medical ICU (2 units) | Primary diagnosis cardiac surgery 2(10%), CHF, MI, endocarditis 4(20), pneumonia, asthma, respiratory distress 5(25), non-neurological trauma 2(10%), sepsis 2(10%), Altered mental status 3(15%), liver failure, hyperglycaemia 2(10%).  n=20, M 10(50%),  mean age 50.5 years, range 25-80 (sd 16).  Ethnicity white not Hispanic 11(55), black, not Hispanic 9(45).  Endotracheal intubation yes 12(60%), no 8(40%). | Convenience sample of 20. Inclusion: >18 years old admitted to the medical respiratory and coronary medical ICU's during an 8 month period (October 2001-May 2002). Presence of indwelling arterial catheter was required for continuous monitoring of arterial blood pressure.  Exclusion: patients who were receiving neuromuscular blocking agents and those with overt disease affecting the brain (e.g. head trauma, intracranial haemorrhage, meningitis, stroke). | Activity counts | No |  |
| 12 | Lehmkuhl *et al*., 2022, Denmark  Daily variation in physical activity during mechanical ventilation and stay in the intensive care unit.  Prospective observational study | ICU - MV patients from 2 mixed ICUs (university hospital and regional hospital in Denmark). | Admission diagnosis respiratory 17(44), CV 4(10), sepsis 11(28), GI 7(18),  n=39, 19 female (49%),  age 69yrs median (IQR 62-77),  BMI NR, Ethnicity NR,  mechanical ventilation 8 days (IQR 4-19) | Inclusion: patients admitted to ICU between sept 2107-April 2018 and October 2019 and January 2020. Aged 18 years or older, required ICU treatment for more than 24hr and expected to be on MV.  Exclusion: patients categorised as unstable or inevitably dying, patients with cognitive impairment (diagnosed with dementia, autism or mentally retarded), inability to speak and understand Danish or give consent. | Time in activity | No |  |
| 13 | Rollinson *et al*., 2022, Australia  Physical activity of patients with critical illness undergoing rehabilitation in intensive care and on the acute ward: An observational cohort study  Prospective observational study | 24 bed ICU | ICU diagnosis cardiovascular 9(15%), respiratory 13(22%) sepsis(nonpulmonary) 2(3%), liver failure 20(34%), GI 7(12%), other 8(14%) n=59, Male 34(58%),  Age 60+14,  BMI NR,  Ethnicity NR,  Duration of mechanical ventilation 7 days (5-14). | Inclusion: Enrolled between November 2014 and September 2016, adults >18 years old with critical illness who received more than 48h of invasive mechanical ventilation in the ICU and expected to stay in ICU for more than 4 days, were awake and able to follow instructions and considered ready to commence rehabilitation according to established safety criteria.  Exclusion: had a primary neurological diagnosis, had a primary neuromuscular insult, were unable to mobilise for more than 15m premorbidly according to the medical record, had a medical contraindication to mobilisation, were included in alternate studies involving novel approaches to early rehab or were previously enrolled in this study. | Time in activity,  activity intensity | No |  |
| 14 | Schujmann *et al*., 2019, Brazil  Impact of a progressive mobility programme on the functional status, respiratory and muscular systems of ICU patients: A randomized and controlled trial.  RCT | ICU | n=67 control group, n=68 intervention.  Female 23(46) control, 27(54) intervention;  days of mechanical ventilation 2.5(1-4) control, 2 (1-3) intervention;  Mechanical ventilation 17(34) control, 23(46) intervention.  BMI NR,  Ethnicity NR. | Inclusion: >18yrs, scoring 100 points on the Barthel Index (BI) in the 2 weeks prior ICU admission. Adults with previous functional independence and without contraindications for mobilisation.  Exclusion: previously hospitalised in other hospitals, neurologic alterations, stayed less than 4 days in ICU, they were amputees on admission, had contraindications for mobilisation, had cognitive impairment with an inability to understand commands and perform tests. | Step counts,  activity intensity | Yes |  |
| 15 | Elias *et al.,* 2021 a, USA  Graphic representation of hourly activity counts may identify discharge outcomes for older adults after critical illness.  Prospective exploratory research design | Ward-medical/surgical, within 24-48hr ICU discharge | Primary ICU admission: Medical 12(42.9%), surgical CV 10(35.7%), surgical transplant 2(7.1%), trauma 2(7.1%), neuroscience 2(7.1%).  n=28. Female 11(39.3%),  Age 71.1+4.6,  Race/ethnicity: White and non Hispanic/Latino 21(75%), Black/African American 4(14.3%), Hispanic/Latino 3(10.7%).  Duration of mechanical ventilation 5.4days+7.6.  BMI not reported. | Inclusion: community dwelling (admitted from home) older adults >65yrs, recently transferred out of ICU (within 24-48 hours), mechanically ventilated while in ICU, functionally independent prior to hospitalisation. Katz Index of Activities of Daily living of 6 or more.  Exclusion: pre-existing diagnosis of mild cognitive impairment or dementia, history of psychiatric disorder, active palliative care or hospice orders, and/or spinal cord injury, those admitted from care facility or received home health care at home prior to admission. | Activity counts | N/A |  |
| 16 | Elias *et al.,* 2021 b, USA  Daytime activity and sleep are associated with motor function in older intensive care unit survivors.  Cross-sectional | Ward-medical/surgical, within 24-48hr ICU discharge | n=30, 63.3% male mean age 71.4+5.4yrs, BMI NR,  76.7% identified as white and non-Hispanic Latino.  Average length of mechanical ventilation about 5 days.  About 43.3% were discharged to a skilled nursing facility or long term acute care facility. 1 participant readmitted and didn't complete full actigraphy observation period, 3 participants cold not complete at least one of the motor function assessments. | Inclusion: ICU survivors, >65yrs, functionally independent prior to hospital admission (Katz index 6 or more), mechanically ventilated in ICU and recruited within 24-48 hours of ICU discharge. Community dwelling (admitted from home) prior to hospital admission. | Activity counts | N/A |  |
| 17 | Elias *et al*., 2021 c, USA  Daytime activity is associated with discharge to home in older adults recovering from critical illness.  Prospective exploratory research design | Ward-medical/surgical, within 24-48hr ICU discharge | n=30. Male 19(63.3%) female 11 (36.7%)  Age 71.37yrs (sd 5.35, range 65-86),  BMI NR,  Race/ethnicity: White 26(86.7%), black or African American 4(13.3%), Hispanic or Latino 3(10%),  Length of mechanical ventilation 5.18 days (sd 7.4, range 0.5-36) | Inclusion: 30 English-speaking hospitalised older adults (aged>65 years), functionally independent prior to hospitalisation, mechanically ventilated while in ICU, recently transferred out of ICU (within 24-48 hours post ICU discharge).  Exclusion: pre-existing diagnosis of dementia, imminent death, active palliative care or hospice orders, and/or spinal cord injury. Individuals admitted from a long-term care facility, assisted living facility or skilled nursing/rehab facility or those who received private/home health care at home prior to admission were excluded from the study. | Activity counts | N/A |  |
| 18 | Gupta *et al*., 2020, USA  Use of actigraphy to characterize inactivity and activity in patients in a medical ICU  Prospective observational study | ICU | N=34; Female 17 (50%);  Age median 60, IQR 44,69;  BMI <18 3(9%), normal 18-24.9 13(38%), overweight 25-29.9 9(26%), obese >30 9(26%);  Non-Hispanic White Race 21 (64%);  ICU admission diagnosis category respiratory failure 14(41%); GI 3 (9%), sepsis 7(21%), CV 4 (12%), other 6 (18%). ? duration of mechanical ventilation - 11(32%) received mechanical ventilation. | Inclusion: Aged 18 years and older.  Exclusion: Moribund, awaiting transfer out of ICU, awaiting for procedures involving the wrist, no available wrist (e.g. due to lines placed in hand/arm), or unable to provide informed consent in English. | Activity counts | N/A |  |
| 19 | Winkelman, 2010, USA  Investigating activity in hospitalised patients with chronic obstructive pulmonary disease: A pilot study.  Observational, exploratory study | ICU and step down unit | N=17, Female 14 (82%);  Age mean: 60 (range 35-74) SD 8.8;  BMI: 38 (15.8-87.3) SD 17.0;  Reason for admission: COPD exacerbation; 15, COPD exacerbation with pneumonia; 2  All subjects MV (invasively or noninvasively ) | Patients with diagnosis of "COPD exacerbation" were included. P/F ratio 0f 100/40, Fi02 > 0.6, First observation must be between 48-60 hours of admission to the unit. Patients were excluded if they had multiple primary diagnoses. | Activity counts | No control |  |
| 20 | Beach *et al.,* 2017, Australia  Measurement of physical activity levels in the intensive care unit and functional outcomes: An observational study  Prospective, non-interventional Observational study | ICU | N=60, Male: 33 (55%);  Reason for admission: Medical: 33 (55%), Surgical 20 (33%), Trauma 7 (12%);  Diagnosis: Cardiac arrest 14 (23%), Pneumonia 8 (13%), trauma 7 (12%), cardiovascular surgery 7 (12%), gastrointestinal surgical 7 (12%), Gastrointestinal non-surgical 6 (10%), Sepsis 4 (7%), endocarditis 2 (3%), other 5 (8%).  Median MV time days: 4.0 [IQR 3.0-8.0]  BMI: NR,  ethnicity: NR. | Inclusion: Adults 18 years or older, MV within 48 hours of admission, MV for at least 48 hours, predicted to remain in ICU for 5 days.  Exclusion: Unable to mobilise due to major trauma necessitating a specific period of immobilisation, new neurological issue such as stroke, traumatic brain injury, or spinal cord injury and poor premorbid mobility. Non English speaking or non Australian citizens. | Step counts,  time in activity | No control |  |
| 21 | Beach *et al.,* 2014, Australia  Low physical activity levels and poorer muscle strength are associated with reduced physical function at intensive care unit discharge: An observational study.  Prospective observational study | ICU | N=48, Male: (54.4%);  Mean age 59.5 (SD 15.4);  Reason for admission: Medical 25 (54.4%), emergency surgical, 17 (37%), Elective surgical, 4 (8.7%);  Duration of intubation, (days) median 5.0 [IOR 3.0-8.0];  BMI: NR;  Ethnicity: NR | NR | Step counts,  time in activity | NR |  |
| 22 | Estrup *et al.,* 2018, Denmark  Physical function and actigraphy in intensive care survivors—A prospective 3‐month follow‐up cohort study | Hospital ward to 3 months post discharge | N=44; Male n=26 (59%) Mean age: 72 (SD 10) Admission type: Surgical n=16 (36%) BMI, mean 28 (SD 8) ICU LOS; median days 4 (2-7) | All patients who were deemed eligible for the original study. Estrup S, Kjer C, Vilhelmsen F, Poulsen LM, Gøgenur I, Mathiesen  O. Cognitive function 3 and 12 months after ICU discharge—a prospective cohort study. Crit Care Med. 2018;46(12):e1121‐e1127.  Exclusion: Any patients with total paralysis or no ambulatory function. | Activity counts | No control |  |
|  | Abbreviations: N/R: not recorded or stated; N/A: Not applicable; 6MWD: 6 minute walk (test) distance; MIP: Maximal inspiratory pressure; PA: Physical activity; SB: Sedentary behaviour; MV: Mechanical ventilation; Fi02: Fraction of inspired oxygen; P/F ratio: Pa02/Fi02 | | | | | |  |
|  |  |  |  |  |  |  |  |
