# Supplementary material for: Device-based measurement of physical activity and sedentary behaviour after critical illness: A scoping review
Source: PLoS One. 2025 Jun 3;20(6):e0322339. doi: 10.1371/journal.pone.0322339 (PMC12133016; doi:10.1371/journal.pone.0322339)
Supplement: S3 Table — (DOCX) [file pone.0322339.s003.docx]

| **Table S3 Correlations device measured physical activity and other health outcomes measured** | | | | | | |
| --- | --- | --- | --- | --- | --- | --- |
| ***Author, year*** | ***Other physical activity measures*** | ***Exercise Capacity*** | ***Muscle strength*** | ***HRQOL*** | ***Hospital LOS*** | ***Other*** |
| Baldwin et al. 2020 | Better physical function on the PFIT-s and DEMMI at awakening (T1) was associated with less time (%) spent lying/sitting (rho = 0.608 and = 0.639 respectively, both p < 0.001)  More minutes spent upright (rho = 0.608 and 0.639, respectively, both p < 0.001), and more sit-to-stand transitions (rho = 0.539 and 0.626, respectively, both p < 0.001) Similarly at ICU discharge, better PFIT-s and DEMMI scores were associated with less time (%) spent lying sitting (rho = 0.810 and = 0.732, respectively, both p < 0.001)  More minutes spent upright (rho = 0.809 and = 0.730, respectively, both p < 0.0001).  More sit-to-stand transitions (rho 0.781 and 0.730, respectively, both p <0.001) There were no associations between sedentary variables and physical function at hospital discharge. | NR | The pattern of associations between sedentary variables and muscle strength was similar; less % time spent lying/sitting, more minutes spent upright, and more sit-to-stand transitions were associated with better muscle strength (MRC-ss) at awakening and better muscle strength (MRC-ss and handgrip) at ICU discharge. There were no associations between sedentary variables and muscle strength at hospital discharge. There were no associations between physical activity variables with strength or function at any time point. | NR | There were no associations between sedentary or activity variables at any time point with hospital LOS | NR |
| Gandotra et al.  2021 | Correlation of SPPB Scores at ICU and hospital discharge with physical activity - Spearman model.   SPPB scores at ICU discharge and hospital discharge correlated with PA during first 3 days in the community. (r=0.54, p=0.49 and r=0.60, p=0.27) | NR | NR | NR | NR | NR |
| Mc Nelly et al 2016 | NR | NR | NR | ICU survivors had significantly worse PCS and PF compared with controls (41+12 Vs 50+10, p<0.001 and 52+36 Vs 88+20, p<0.007).  Significant differences were seen between previously healthy ICU survivors and those with chronic disease, in PCS (46, 95%CI 39.9-52.0) Vs 34 28.0-40) p=0.007 and PF scores 68.4 (50.1-86.8) Vs 29.1 (12.4-45.7) p=0.003.  Calculations were performed for normalisation of SF-36 PCS for patients without pre-morbid chronic disease and those from the whole survivor group (score of 50). | NR | Frailty = median clinical frailty score (CFS) was high in ICU survivors than sex and aged matched controls. Differences were also seen between previously healthy and chronic disease cohorts (2.0 IQR 2.0-4.8) Vs 5.0 (4.0-7.0) respectively; the latter sub cohort had a higher median CFS score than the matched controls 2.0 (1.0-3.0). Frailty = a CFS score of 3 indicates low PA in a non-frail population (projected level for those with pre-morbid chronic disease), a score of 2 indicates normal activity (projected level for those without pre-morbid chronic disease.   Correlation between CFS and daily step count r2 0.55 |
| Van Bakel et al. 2022 | NR | NR | NR | NR | NR | PA levels and SB were not impacted by patient characteristics, disease characteristics, cardiac dysfunction or persistence of symptoms post discharge. |
| Plekhanova et al. 2022 | NR | NR | NR | NR | NR | Those with the most severe acute illness had 1-2mg lower volume of physical activity (p=0.045) and less time spent in MVPA (p=0.032) Women who received IMV had the lowest levels of MVPA (13.7 min/day; 95% CI 7.3, 20.2)  Lower severity of symptoms (excluding cognition and anxiety) were positively associated with PA (p=<0.05) |
| Camus-Molina et al. 2020, | No correlations were found between activity counts, or activity time as measured by the accelerometer and the FSS-ICU, however, inactivity time was negatively associated with mobility at awakening (rho = -0.62, p<.001) and ICU discharge (rho= -0.79, p<.001) | NR | NR | NR | NR | NR |
| Grap et al. 2005, USA | NR | NR | NR | NR | NR | Activity counts taken from wrist actigraphy correlated (r=0.58) with scores on the Richmond agitation-sedation scale (RASS), correlation with ankle actigraphy and RASS (r=0.52) was reported to not be as robust |
| Rollinson et al. 2022, Australia | Change in mean daily PA from ICU to ward was not correlated with physical function, p=0.527. | An increase in the duration of PA in ICU was strongly associated with an increase in PA on the ward (r = 0.728 p<0.001), an extra minute of PA in ICU = 1.6 times the exercise in the ward. | NR | NR | NR | MRC sum score (MRC-SS). Range from 0 (complete paralysis) to 60 (normal strength). 29(49%) were diagnosed with ICU-AW on awakening. Participants with ICU-AW on awakening were less likely to be discharged directly home (chi-squared = 8.97, p=0.030 |
| Schujmann et al. 2019, Brazil | Higher scores on the (BI) were shown in the intervention group undertaking a progressive mobility programme when compared to the control group (97±5 vs 76±20 p <0.001) higher scores on the BI were associated with increased PA levels in the ICU | NR | NR | NR | Shorter ICU stays were shown in the IG (p=0.003) | TUG (timed up and go test) to evaluate mobility. Functional status using BI and the ICU mobility scale. A higher BI was associated with the amount of exercise |
| Elias et al. 2021, | NR | NR | NR | NR | NR | Discharge deposition to home (minimal assistance or home with home health care) Vs discharge to a facility (inpatient rehab facility, skilled nursing facility or long-term acute care hospital). discharged home: 2,862+908 activity counts/hr compared to discharged to inpatient rehab facility 2062+933 activity counts/hr), long term acute care hospital (478+444 activity counts/hr) |
| Elias et al 2021b | Motor battery assessed grip strength and dexterity (9-hole pegboard dexterity test) | NA | The regression model exploring the relationship between post-ICU daytime activity and grip strength was significant (R^2^ =0.689 p<0.001),  daytime activity counts positively correlated with grip strength (B=0.258, p=0.035), indicating lower daytime activity was associated with worse performance on the grip strength test. | NA | NR | The regression model exploring the relationship between post-ICU daytime activity and dexterity was significant (R2=0.453, p<0.019), lower daytime activity counts were associated with a greater/longer time in seconds to complete 9-hole pegboard dexterity test (B=-0.376, p=0.037), indicating that lower daytime activity was associated with worse performance on dexterity. |
| Elias et al 2021c | NA | NA | NA | NA | The regression model exploring the relationship between post-ICU daytime activity and LOS was significant (R2=0.708, p<0.001).  Post-ICU daytime activity was negatively associated with hospital LOS (b=-0.322, p=0.041) after adjusting for covariates. The unique variance for post-ICU daytime activity was 7.84%.  Lower post-ICU daytime activity was associated with longer LOS.  Post-ICU nighttime activity was not significantly associated with hospital LOS (b=-0.279, p=0.066). | n=28 with available discharge data, significant differences in post-ICU daytime activity by discharge disposition t[26]=2.054, p=0.050).  Post-ICU daytime activity was greater among those discharged home (54.42 activity counts/min (sd 29.3, range 5.8-89.5) than those discharged to a facility (33.26 activity counts/min, sd 24.26 range 7.7 to 91.2).  There were no significant differences in nighttime activity by discharge disposition. Discharge to home (i.e., home with minimal assistance or home with home health care, facility (inpatient rehabilitation facility, skilled nursing facility, long-term acute care hospital). |
| Beach et al 2017 | Fair-to-moderate correlation between PA duration (minutes) and PFIT-s at ICU discharge r = 0.4 (p=0.01) | NR | NR | NR | NR | Correlation between active energy expenditure (SWA-MF) and the highest level of mobility (IMS) on day 5 (r=0.76, p=0.00) |
| Beach et al. 2014 | Good correlation between PA level and physical function (rho=0.707, p<0.005) | NR | NR | NR | No relationship between PA levels and ICU/hospital LOS. | Moderate correlation between sedation score and PA at day 5, (rho=0.601, p<0.005). |
| Estrup et al 2018 | CPAx at ICU discharge to activity, found significant correlations for mean daily activity (R2 = 0.14, P = 0.017),  mean daytime activity (R2 = 0.12, P = 0.030),  maximum activity on second day (R2 = 0.18, P = 0.0053), total activity in daytime on day 2 (R2 = 0.18, P = 0.0058).  This confirms a relationship between the physical function measured by CPAx and the activity of the patient. | NR | NR | NR | NR | Higher activity levels the first week after ICU discharge did not predict a higher gain in physical function   The correlations were as follows:  Mean activity per day to CPAx at ICU discharge, R2 = 0.12, P = 0.14; CPAx at 3 months, R2 = 0.20, P = 0.058; ∆‐CPAx (ICU‐discharge to 3 months), R2 = 0.25, P = 0.028 (Figure 3). Mean activity level during daytime was significantly correlated to CPAx at 3 months, R2 = 0.21, P = 0.047, but not to CPAx at ICU discharge or ∆‐CPAx |
| Abbreviations: PFIT: Physical Function in Intensive Care Test,DEMMI; de Morton Mobility Index DEMMI: SPPB: Short Performance Physical Activity Battery, BI Barthel Index, CPAx: Chelsea Critical Care Physical Assessment Tool, ICU-AW: Intensive Care Unit Acquired Weakness, CFS: Clinical Frailty Score, MRC-SS: Medical Research Council Sum Score, | | | | | | |
