# Supplementary material for: Device-based measurement of physical activity and sedentary behaviour after critical illness: A scoping review
Source: PLoS One. 2025 Jun 3;20(6):e0322339. doi: 10.1371/journal.pone.0322339 (PMC12133016; doi:10.1371/journal.pone.0322339)
Supplement: Attachment — Submitted filename: pone.0322339.docx [file pone.0322339.s007.docx]

**Full title: Device-based measurement of physical activity and sedentary behaviour after critical illness: A Scoping Review**

**Darren Murphy^1^*, Annette Henderson^2^, Judy M Bradley^3^, Bronwen Connolly^3^, Jason J Wilson^4¶^, Brenda O’Neill^1^*^¶^**

**^1^Institute of Nursing and Health Research, School of Health Sciences, Ulster University, Belfast, UK**

**^2^School of Health Science, Derry / Londonderry campus, Ulster University, Derry / Londonderry, UK**

**^3^Wellcome-Wolfson Institute for Experimental Medicine, School of Medicine, Dentistry and Biomedical Sciences, Queen’s University, Belfast, UK**

**^4^School of Sport and Exercise Science, Derry / Londonderry campus, Ulster University, Derry / Londonderry, UK**

*** Corresponding author(s)**

**E-mail:** [**murphy-d76@ulster.ac.uk**](mailto:murphy-d76@ulster.ac.uk) **(DM)**

[**b.oneill@ulster.ac.uk**](mailto:b.oneill@ulster.ac.uk) **(BON)**

**^¶^ Joint senior authors**

**Short title**: Device-based physical activity after critical illness: A scoping review

# Abstract

Introduction: Measuring and promoting physical activity could support rehabilitation and recovery after critical illness. In recent years, there has been an emergence of the use of devices to measure both physical activity and sedentary behaviour in this population. Understanding device selection and processes for data analysis could be helpful for future research and practice when used with this population.

Aims: The aim of this review was to explore the current use of device-based physical activity instruments to measure physical activity and sedentary behaviour during and following critical illness.

Methods: A scoping review was conducted which followed the Arksey and O’Malley (2005) framework. A comprehensive search of four electronic databases (Medline, ProQuest, Scopus and CINAHL) was conducted using pre-agreed search terms. Screening and data extraction was conducted by two independent reviewers. Data were analysed descriptively by summarising and describing results that linked to the review questions.

Results: Twenty-two studies were included; the majority were observational (n=12), with one randomised control trial. Studies covered the continuum from intensive care admission to 18 months post-hospital discharge. A total of 11 devices were used to assess physical activity and sedentary behaviour, and many different processing decisions were used for data analysis. Physical activity levels were low in the intensive care unit and remained low following discharge from intensive care.

Conclusion: The use of device-based measurement of physical activity and sedentary behaviour after critical illness is an emerging research area. While several devices are available, this review highlights the need for agreed and standardised protocol(s) to guide the processing and analysis of data. Investment is also needed to support the recovery of physical activity and the reduction of excessive sedentary behaviour following discharge from the hospital.

# Introduction

After critical illness, reduced function, mobility, and strength may negatively impact quality of life and capacity to perform routine daily tasks [1-3]. In addition, patients often have difficulty achieving pre-illness physical activity levels [4]. These functional limitations are likely to prevent survivors of critical illness from achieving the recommended weekly levels of physical activity. Adults are recommended to undertake ≥150 mins of moderate intensity, ≥75 minutes of vigorous-intensity physical activity, or a suitable combination of both [5]. Guidelines also recommend a reduction in sedentary behaviour [5]. The effects of high levels of sedentary behaviour have been well documented, with sedentary behaviour having negative effects on health and increasing the risk of mortality in older adults [6,7]. Negative physiological effects of sustained levels of sedentary behaviour include insulin resistance, vascular dysfunction and a reduction in muscle mass and strength [8]. Reallocation of the length of time spent in sedentary behaviour into moderate to vigorous physical activity (MVPA) can result in health benefits, with researchers reporting that changes of as little as 4-12 minutes can be useful [9]. The consequences of critical illness, as well as excessive sedentary behaviour, have the potential to impact patients’ recovery and return to activity after hospital discharge. However, physical activity is not routinely measured in this population, and it is not clear which device/s should be used [10]. There is also limited data to quantify and describe levels of physical activity and sedentary behaviour after critical illness.

There is increasing awareness about the need to evaluate and promote rehabilitation and physical activity in the critical care population during and after hospitalisation [11]. A previous scoping review (n=7 studies) aimed to evaluate the use of wearable devices in the intensive care unit (ICU) and found that device-based physical activity measurement was used infrequently in people after critical illness [12]. However, this is an emerging area of research and several papers about physical activity after critical illness have been published since the Gluck *at al*. (2017) review [13,14]. Researchers and clinicians will need to be able to accurately assess physical activity and sedentary behaviour levels to identify activity patterns across the trajectory of recovery, evaluate the prevalence of physical activity engagement, and assess the effectiveness of interventions designed to change physical activity and sedentary behaviour. It is anticipated that this review will advance knowledge about device-based instruments, and any challenges for the measurement of physical activity and sedentary behaviour in this population. This review will inform the potential use and implementation of device-based measurement of physical activity and sedentary behaviour following critical illness in future research and in clinical practice.

## Aims

This scoping review aimed to explore the current use of device-based physical activity instruments to measure physical activity and sedentary behaviour during and following critical illness. The objectives were, in patients following critical illness, to identify device-based instruments and the parameters used to describe patterns and levels of physical activity and sedentary behaviour, report device clinimetric properties; describe the relationship between physical activity and sedentary behaviour with other health-related outcomes and explore the levels and patterns of physical activity and sedentary behaviour across the trajectory of recovery.

# Methods

We chose a scoping review as the most appropriate review method in order to synthesise the evidence to address specific questions relating to the device-based measurement of physical activity and sedentary behaviour [15]. The scoping review applied the framework by Arksey and O’Malley [16], which was further refined by Levac [17]. The methodology is described in the study protocol (published in advance <https://osf.io/278mc/>) and is reported according to the Preferred Reporting Items for Systematic reviews and Meta-Analyses extension for Scoping Reviews (PRISMA-ScR) [18]. There was one deviation from the published protocol as two electronic databases, PubMed and Embase, were removed based on a recommendation from the subject librarian and were replaced with Scopus and ProQuest. This scoping review aimed to address the following questions:

1.	What device-based instruments are currently used to measure physical activity and sedentary behaviour during and following critical illness?

2.	What parameters have been used to describe patterns and levels of physical activity and sedentary behaviour in patients following critical illness?

3.	What are the clinimetric properties (validity, reliability, responsiveness), user acceptability, and minimal clinically important differences (MCIDs) of device-based instruments to measure physical activity and sedentary behaviour specifically in patients following critical illness?

4.	What is the relationship between physical activity and sedentary behaviour with other health-related outcomes (e.g., quality of life, hospital readmission, exercise capacity, function and emotional status) in patients following critical illness?

5.	What are the levels and patterns of physical activity and sedentary behaviour in patients following critical illness across the trajectory of recovery?

## Search Strategy

The research team collaborated with a research librarian to develop the search strategy, including the selection of the databases and refining the keywords and phrases. A comprehensive search of four electronic databases (Medline, ProQuest, Scopus and CINAHL) was conducted using the agreed-upon search terms. Medical subject headings (MeSh) terms were expanded and included in the relevant databases (Supplement 1). The research team met to provide their experience and methodological expertise to further guide the identification of relevant studies.

Results were searched from inception to December 2023 and were limited to human research and English language.

## Eligibility criteria

Studies which reported the outcome of physical activity or sedentary behaviour in survivors of critical illness or ICU were included. The full inclusion criteria were as follows:

1.	Adult patients admitted to an ICU (≥18 years old).

2.	Patients who have been mechanically ventilated in ICU.

3.	Critically ill patients previously admitted to an ICU e.g. medical, surgical, neurological, cardiac and trauma diagnoses.

4. Physical activity monitoring that had commenced: during ICU stay, post-ICU discharge (on the ward) or post-hospital discharge.

5. Included some form of device-based physical activity measurement used to assess “physical activity” and/or “sedentary behaviour”, including but not limited to accelerometers, pedometers, activPAL, ActiGraph, smartphone apps etc.

## Study selection

The search results from each of the databases were imported into Covidence, which was used as a screening and data extraction tool. Duplicates were removed automatically. Each study title and abstract from the systematic search was screened by two independent reviewers [DM and AH] against the inclusion and exclusion criteria. The full text was retrieved for all of those that appeared to meet the inclusion criteria, and for those where there was insufficient information regarding whether the criteria were met.

All full-text papers were then independently reviewed by the two reviewers [DM and AH] to determine inclusion. Any disagreements were addressed via discussions, or with the consultation of a third reviewer [BON or JW] when required.

## Data extraction and analysis

The studies were randomly divided between the two reviewers for completion of data extraction (DM & AH). The data were transferred to tables on an Excel database, which was drafted specifically to capture all the information required to answer the research questions. The data extracted included study characteristics, device details, parameters of physical activity and sedentary behaviour, correlations with other outcomes and levels of physical activity and sedentary behaviour. Once completed, the tables were reviewed by DM and AH to ensure all relevant data were captured and collated. Data were analysed descriptively by summarising and describing results that linked to the review questions. Thorough discussion took place among the review team to agree all results

# Results

## Study selection

The identification and selection of studies is summarised in Figure 1. There were 22 studies included.

Figure 1. Prisma flow diagram for selection of studies [19]

## Study characteristics

Study characteristics are summarised in Table 1 (with further details in Supplemental Table S2). The studies were undertaken in Europe (n=6), North America (n=8), South America (n=3) and Oceania (n=5). There was heterogeneity in the study designs utilised; the majority (n=12) were observational studies, with one randomised control trial [20], and one conference abstract [21] included. Studies collected data across the continuum of recovery, with the earliest timepoint occurring at awakening in ICU [13,22] through to 18 months post-hospital discharge [23]. The years of publication were 2005-2022.

| **Table 1 Physical activity devices including details of wear time, activity parameters and data processing per device** | | | | |
| --- | --- | --- | --- | --- |
| ***Author, year*, aims,** | ***Device, Location worn, Clinimetric properties.*** | ***Wear time.* *When to wear/not to wear*** | ***Activity Parameters*** | ***Device processing parameters*** |
| Grap *et al.,* 2005 [30[.  To determine the feasibility of continuous measurement of limb movement via wrist and ankle actigraphy in critically ill patients and to compare actigraphy measurements with observed activity, subjective scores on sedation-agitation scales and heart rate and blood pressure of patients. | Device: Mini Mitter, Actiwatch  No of axis: Uni-axial  Location worn: Placed on non-dominant wrist and right ankle.  Clinimetric properties: N/A | Worn for the two-hour data collection period | NR | Epoch length: 15 sec  Sampling frequency: NR |
| Winkelman, 2010 [29].  This study investigated whether selected cytokines vary between rest and activity periods in critically ill COPD patients. The secondary aims were to explore the types and duration of brief episodic activity. | Device: Mini Mitter, Actiwatch  No of axis: Uni-axial  Location worn: Worn on the dominant wrist.  Clinimetric properties: N/A | 24-48 hours, beginning with enrolment in the study | Time (duration) of movement as well as intensity | Epoch length: 1 minute  Sampling frequency: NR |
| Beach *et al.,* 2014 [21].  To describe PA levels during ICU admission and barriers to PA, to describe the relationship between PA, muscle strength and physical function at ICU discharge, to describe the relationship between muscle strength, physical function and ventilator-free days and between PA levels and ICU/hospital LOS. | NR | NR | NR | NR |
| Borges *et al*., 2015 [24].  The aim of the study was to quantify (Physical activity in Daily Life) PADL, muscle strength and exercise capacity in short and medium-term survivors of severe sepsis and shock. | Device: Dynaport minimod  No of axis: Tri-axial  Location worn: lower back near L2.  Clinimetric properties: N/A | Wear for 2 consecutive days | Time spent, sitting, lying, standing, and walking, changes in body position and energy expenditure. | Epoch length: NR  Sampling frequency: NR |
| Mc Nelly *et al.,* 2016 [23].  To explore the relationship between objective measures of PA, self-reported measures of physical HRQOL, and clinician-reported global functioning (frailty). To investigate the relationship between chronic disease status prior to critical illness and functional outcome | Device: SenseWear armband  No of axis: Bi-axial  Location worn: Upper arm, not stated which side.  Clinimetric properties: Validity and acceptability | Wear daily for 5 days to include 1 WE day, valid day ≥90% on body time per day for ≥ 5 days. , | Number of steps, HRQoL, clinical frailty score | Epoch length: NR  Sampling frequency: NR |
| Beach *et al.,* 2017 [22].  Quantify levels of PA and evaluate the correlation of SWA-MF measurement of active and resting energy expenditure against the ICU mobility scale. Secondary outcomes, describe the feasibility of using the SWA-MF in the ICU setting, describe changes in physical function over the ICU stay and the relationship between PA duration and physical function. | Device: SenseWear armband (SWA-MF)  No of axis: Bi-axial  Location worn: Posterior aspect of the mid upper arm, Dominant/non dominant not reported.  Clinimetric properties: Acceptability reported | 24 hours per day for 5 days, valid day, minimum of 8 hours data for 3 consecutive days. | PA was defined as > 1 MET, number of steps, total energy expenditure, and active energy expenditure. | Epoch length: 1 minute  Sampling frequency: NR |
| Baldwin *et al.,* 2018 [26].  To test the hypothesis that there would be an agreement between the Activ Pal accelerometer and direct observation in classifying sit-to-stand transitions, time spent standing, stepping, upright and sedentary | Device: ActivPAL 3  No of axis: Tri-axial  Location worn: Thigh, worn on the non-dominant side.  Clinimetric properties: Validity assessed | NR | Time spent, sitting, standing, or stepping. | Epoch length: NR  Sampling frequency: NR |
| Estrup *et al.,* 2018 [28].  The primary outcome was the association between physical function 3 months after discharge from ICU measured by CPAx and activity levels during the first week at the ward measured by actigraphy. | Device: Micro sleep watch  No of axis: NR  Location worn: Wrist, side not reported.  Clinimetric properties: N/A | Worn for 7 days after ICU discharge | Physical activity was analysed in several ways: as a mean of activity counts per day; as the mean activity per hour during daytime (7 am‐4 pm); as a mean difference between day and night; as the maximum activity in 1 hour on day 2; as total activity count on day 2; and finally, as daytime activity on day 2 | Epoch length: NR  Sampling frequency: NR |
| Anderson *et al.,* 2019 [31].  Investigate the validity of the Actigraph GT3X accelerometer, quantify step count during self-selected distances and walking speeds in ward-based hospital patients recovering from critical illness | Device: Actigraph GT3X  No of axis: Tri-axial  Location worn: One is attached to the anteromedial thigh and the other to the lateral aspect of the ankle (above lateral malleolus). Both non-dominant leg.  Clinimetric properties: Validity assessed | For the walking aspect of the protocol. | Observed step count was compared to step count data captured by ankle and thigh-mounted accelerometer | Epoch length: NR  Sampling frequency: NR |
| Schujmann *et al.,* 2019 [20].  The aim was to investigate whether patients who participated in a mobility program in the ICU performed better on functional status, muscle, mobility, and respiratory assessments upon discharge than patients who received conventional physiotherapy. | Device: Actigraph GT3X  No of axis: Tri-axial  Location worn: Placed on the dominant lower limb.  Clinimetric properties: N/A | Time spent in ICU. The accelerometer was not removed until ICU discharge. | Amount of inactive time and total time in levels of light and moderate intensity of activity. | Epoch length: NR  Sampling frequency: NR |
| Baldwin *et al*., 2020 [13].  To examine the patterns of sedentary behaviour and physical activity in people during their hospital-based recovery from a critical illness. | Device: ActivPAL 3  No of axis: Tri-axial)  Location worn: Thigh, worn on the non-dominant side.  Device: GENEActiv  No of axis: Tri-axial  Location worn: Wrist worn on the non-dominant side.  Clinimetric properties: N/A | Wear length: 1 day  Wear / non-wear instructions: Worn continuously except during any off-unit/ward procedures or imaging.  Invalid day if ≥6 h of non-wear. | ActivPAL: % time in sedentary behaviour; time spent upright (minutes); number of upright bouts ≥2 and ≥5 minutes; number of sit-to-stand transitions; number of sitting/lying bouts ≥30 minutes and ≥2 hours)  GENEActiv: % time spent inactive; time spent being active (minutes) | Epoch length: 60 seconds  Sampling frequency: 20HZ (ActivPal)  Epoch length: 60 seconds Sampling frequency: 50HZ (GENEActiv) |
| Camus-Molina *et al.*, 2020 [36].  Assess construct validity of the Chilean-Spanish version of FSS-ICU using 3 variables measured through continuous actigraphy (activity counts, activity time, inactivity time) in mechanically ventilated patients from their admission to ICU discharge. The secondary aim is to test the correlation of the FSS-ICU with MRC-SS ICU length of stay and duration. | Device: Actigraph GT9X Link  No of axis: Tri-axial  Location worn: Right ankle  Clinimetric properties: N/A | Daily for 24 hours from ICU admission to ICU discharge. The accelerometer was removed only for out-of-room clinical tests (i.e. MRI, CT, surgical procedures). Twice a day, patients' skin condition, battery level and functioning of accelerometers were checked. | Activity counts, activity time, and inactivity time were analysed. | Epoch length: 5 seconds  Sampling frequency: 90 Hz |
| Gupta *et al*., 2020 [27].  Use actigraphy to characterise inactivity and activity in critically ill patients | Device: Actiwatch spectrum  No of axis: Uni-axial  Location worn: Right wrist, or if unavailable (i.e., due to arterial line) left wrist.  Clinimetric properties: N/A | 48 hours in ICU. | Steps not measured. Only epochs in terms of zero and non-zero activity epochs were recorded. | Epoch length: 30-second Sampling frequency: NR |
| Elias *et al.*, 2021 a [37].  Examine trends between post-ICU hourly activity counts and discharge disposition among hospitalised older ICU survivors. (Transitioning from ICU hospitalised older adults are inactive which may affect discharge outcomes) | Device: Actiwatch spectrum  No of axis: Uni-axial  Location worn: Worn on the dominant wrist.  Clinimetric properties: N/A | Early post-ICU hourly activity worn continuously 0:00 am - 23:59pm. | Activity counts are generated for each 15-second epoch. | Epoch length: 15 seconds  Sampling frequency: NR |
| Elias *et al.,* 2021 b [33].  Explore associations between post-ICU activity, sleep/rest and motor function among hospitalised older ICU survivors. (hospitalised older ICU survivors are often inactive and experience sleep disturbances). | Device: Actiwatch spectrum  No of axis: Uni-axial  Location worn; Worn on the dominant wrist.  Clinimetric properties: N/A | Worn post-ICU (24-48hr) continuously for 2 nighttime and 1 daytime data capture. | Activity counts are generated for each 15-second epoch. | Epoch length: 15 seconds  Sampling frequency: NR |
| Elias *et al.,* 2021 c [35].  Explore the relationship between early post-ICU activity, discharge deposition and LOS. | Device: Actiwatch spectrum  No of axis: Uni-axial  Location worn: Worn on the dominant wrist.  Clinimetric properties: N/A | Worn continuously throughout a 2 night/1 day observation period, beginning at the time of study enrolment | Activity counts are generated for each 15-second epoch. | Epoch length: 15 seconds Sampling frequency: NR |
| Gandotra *et al.,* 2021 [14].  To quantify activity levels and functional status in critically ill patients as they transition from ICU to the wards and back to the community. | Device: Tractivity  No of axis: Uni-axial  Location worn: Ankle worn, side not stated.  Clinimetric properties: N/A | At time of enrolment to 1 week after discharge.  (Not stated if 24hr wear or when removed) | Step counts at 4 measurement points, mean number steps: 3 days prior to ICU discharge,  3 days prior to hospital discharge,  1-3 days after hospital discharge,  4-6 days after hospital discharge. | Epoch length: NR  Sampling frequency: NR |
| Munro *et al*., 2021 [38].  To explore nighttime sleep efficiency and total sleep time over 5 consecutive nighttime periods, the secondary objective was to report the daily activity ratio and the daily activity counts over 5 consecutive days. | Device: Actiwatch spectrum  No of axis: Uni-axial  Location worn: Wrist worn on dominant side unless blocked by medical lines etc.  Clinimetric properties: N/A | Wear for 5 consecutive days (120 hours) | Not specified, the study is measuring activity using a daily activity ratio (DAR) and hourly activity counts, definition of activity is not defined. | Epoch length: 15 seconds Sampling frequency: NR |
| Lehmkuhl *et al.,* 2022 [32].  This study aimed to describe the objectively assessed type, quantity and daily variation of physical activity among mechanically ventilated patients while in ICU. | Device: Axivity AX3 accelerometer  No of axis: Tri-axial  Location worn: Worn on the thigh and upper body, right side.  Clinimetric properties: Acceptability reported | Worn from inclusion to ICU discharge, Day considered invalid if <50% wear time per day. | Time spent lying, sitting, moving, in-bed cycling, standing, and walking. | Epoch length: NR  Sampling frequency: 25 Hz |
| Plekhanova *et al.,* 2022 [25].  The study aims to describe accelerometer-assessed physical behaviours in patient’s post-hospitalisation with COVID-19 | Device: GENEActiv  No of axis: Tri-axial  Location worn: Wrist worn on the non-dominant side.  Clinimetric properties: N/A | Wear length, 14 days for 24 hours, valid day ≥ 16 hours per day. | Inactivity, LIPA, MVPA | Epoch length: 5 seconds Sampling frequency: 30 Hz |
| Rollinson *et al.,* 2022 [34].  This study aimed to measure the physical activity of patients with critical illness engaged in rehabilitation in the ICU and on the acute ward and report discharge destination, muscle strength and functional outcomes. | Device: SenseWear armband No of axis: Bi-axial  Location worn: Worn on left upper arm.  Clinimetric properties: N/A | Worn 7 days per week during daylight hours only from 8 am-6 pm until hospital discharge or after 14 days in the acute ward, whichever occurred first. The armband was not waterproof so was removed for showering activities. | PA levels >1.5METS. | Epoch length: NR  Sampling frequency: NR |
| Van Bakel *et al.*, 2022 [39].  The main aim of this study was to objectively assess PA, SB and sleep duration in patients with COVID-19 with moderate to severe illness that required hospitalisation. The measurements took place at 3 and 6 months after discharge. | Device: ActivPAL 3  No of axis: Tri-axial  Location worn: Thigh worn, dominant or non-dominant not stated.  Clinimetric properties: N/A | NR | LIPA, MVPA or SB: seated, reclined or lying. | Epoch length: NR  Sampling frequency:20HZ |
| **Abbreviations: N/A: Not applicable, NR: Not recorded, PA: Physical activity, SB: Sedentary behaviour, LIPA: Light intensity physical activity, MVPA: Moderate to vigorous physical activity, MRC-SS: Medical Research Council Sum Score, CPAx: Chelsea Critical Care Physical Assessment Tool, FSS-ICU: Functional Status Score for the Intensive Care Unit** | | | | |

Several studies (n=14) provided data at one time point only, while six provided data across two time points and two studies provided data at three or more time points. Control or comparator groups were included in four studies [20,23-25].

## Population studied

Supplemental Table S2 summarises the characteristics of this heterogeneous population. Length of stay in the ICU varied from 4 [28] to 21 [31] days, and the duration of mechanical ventilation was reported in most 17/22 (77%) studies. The population sample sizes in the included studies ranged between 8 [26] and 715 [25] participants.

## Device-based instruments used to measure physical activity and sedentary behaviour

Eleven different devices were used across the 22 studies (Table 1). Actiwatch Spectrum (n=5), activPAL3 (n=3) and the SenseWear armband (n=3) were the most commonly used devices. Location worn was dependent on the device used, and when reported the most common was the wrist (n=9), thigh (n=4), and ankle (n=4). Ten studies reported device placement: this was on the dominant (n=6) or non-dominant limb (n=5). Wear-time duration was reported in n=18/22 (81%) studies. Wear-time duration varied depending on the aims of the study, when validation studies were excluded, wear-time ranged from two hours [30] to 14 days [25]. In studies where accelerometry was used for more than one day, for analysis the parameters for a valid day varied from a minimum of 8 hours of data per day [22] to ≥90% of on-body time per day for ≥5 days [23].

The sampling frequencies used varied across studies, with many not reporting this (n=16), while other studies used 20-30Hz (n=4), 50Hz (n=1) and 90Hz (n=1). Epoch length also varied, with the majority of studies not reporting this (n=12), while 60-second (n=4), 5-second (n=2), 15-second (n=2) and 30-second (n=1) epochs were reported in the other studies (Table 1).

## Parameters to describe patterns and levels of physical activity and sedentary behaviour

Various parameters (n=4) have been used to describe the levels of physical activity and sedentary behaviour, including step counts, activity counts, time in activity (total daily time, percentage of time, and number of bouts), and intensity of physical activity (Table 1).

## Clinimetric properties of the device-based instruments

Clinimetric properties were measured in five studies [22,23,26,31,32] (Table 1). Validity was assessed in three studies [23,26,31]. The ActivPAL demonstrated good validity when recording sit-to-stand transitions, time spent standing and sedentary behaviour when compared to direct observation, however, the validity of the ActivPAL to record step counts could not be confirmed [26]. In the hospital ward following ICU discharge, an ankle-placed ActiGraph was valid in measuring step counts, with the intraclass correlation (ICC) equalling 0.99, while step counts were shown to be underestimated using a thigh-worn device [31]. Accelerometry-measured step counts were shown to be a valid method of assessing frailty and health-related quality of life (HRQOL), with no floor or ceiling effect using the SenseWear device [23].

Reliability and responsiveness were not reported in any of the studies. Also, no studies reported on the minimal clinically important difference (MCID) for any metric with physical activity or sedentary behaviour. User acceptability was reported in three studies [22,23,32]. The SenseWear device was well-tolerated by participants [23] with 97% of participants wearing it for five days in the ICU [22]. The Axivity device was piloted tested on five participants to with no adverse effects noted while wearing it [32].

## Association of physical activity and sedentary behaviour with other health-related parameters

Physical activity data were compared to other health-related outcomes in 15/22 studies (Table S3).

### Physical function

Eight studies [13,14,20-23,28,33] showed positive correlations between physical activity levels and physical function. However, Rollinson *et al.* [34] did not show any significant associations. In addition, Baldwin *et al.* [13] showed there was no association between sedentary behaviour and physical function [13].

### Exercise capacity

A strong correlation was reported between physical activity and exercise capacity in the ICU and in the ward (r=0.728; p<0.001) [34].

### Muscle strength

Less time sitting or lying, with more minutes spent upright and an increased number of STS transitions, were associated with better muscle strength (MRC-ss and handgrip) at ICU discharge, however, there were no associations found at hospital discharge [13]. There was a positive association between post-ICU daytime activity and grip strength (r²=0.689, p<0.001) [33].

### HRQOL

Daily step count was shown to correlate with the physical function component of the SF-36 (r²=0.51; p<0.01) and a weaker correlation (r²=0.25; p<0.01) was shown with the physical component score of the SF-36 [23].

### Hospital LOS

Two studies [13] and [21] showed no associations between physical activity and/or sedentary behaviour and hospital length of stay, however, Elias and colleagues showed there was a significant relationship between post-ICU daytime activity and LOS [35].

### Other

Moderate correlations were shown between the Riker Sedation and Agitation scale (SAS) (rho=0.601; p<0.005) and the Richmond Agitation Sedation Scale (RASS) r=0.58 & r=0.52) and physical activity levels [21,30].

Lower levels of symptom severity, excluding cognition and anxiety, were associated with physical activity (p<0.05), with those experiencing the most severe acute illness having 1-2mg lower volume of physical activity (p=0.045) and less time spent in MVPA (p=0.032) [25].

No studies explored the association between physical activity and/or sedentary behaviour with hospital readmissions.

## Levels and patterns of physical activity and sedentary behaviour

Physical activity and sedentary behaviour patterns were reported from awakening in ICU [22,26] up to 18 months post-discharge [23] in Table 2.

| **Table 2: Measured Physical Activity and Sedentary Behaviour** | | | | |
| --- | --- | --- | --- | --- |
| **Study** | **Location** | **Step counts, Activity counts** | **Time in activity** | **Sedentary behaviour** |
| Grap *et al.,* 2005 [30]. | ICU | **Total activity count for 15 min epoch**. **Wrist:** mean 418, sd 591.7, range 0-2903, median 157.5, IQR 11.3,571.3. **Ankle:** mean 147, sd 387.3, range 0-3593, median 38, IQR 3, 132.3 | N/A | N/A |
| Winkelman, 2010 [29]. | ICU and step-down unit | **Mean no of activity counts:**  Day 1: 98,  Day 2: 124 | N/A | N/A |
| Beach *et al.,* 2014 [21]. | ICU | **Steps per day:**  Median 3.5 [IQR 1.4-5.9] | **Duration of PA (minutes) per day:**  Median 9.3 [IQR 0.4-151.2],  METS: 0.9 [IQR 0.8-1.0] | N/A |
| Borges *et al.,* 2015 [24]. | ICU to 3 months post-hospital discharge. | N/A | Walking time per day was reduced in septic patients compared to the healthy control at ICU (1.9 ± 1.6 vs 10.1 ± 4.4 % p < 0.05) and at 3 months (6.3 ± 3.0 vs 10.1 ± 4.4 %. P < 0.05) [Each per cent represents 7.2 min/day]; Sepsis patients had a lower walking intensity compared to control after hospital discharge, (2.1 ± 0.3 vs 2.5 ± 0.4 m/s^2, p <0.05). At 3 months, 65% of Septic patients walked 30 min per day compared to 85% in healthy subjects. | Participants spent 89.2% of their ICU time sitting or lying down. After hospital discharge, this was reduced to 58% |
| McNelly *et al.,* 2016 [23]. | 18 months post-hospital discharge. | **Mean step count**:  5,803 95%CI 4792-6813 was lower than in controls 11735 95%CI 10928-12542 p<0.001) and lower in those with chronic pre-existing chronic disease 2989 95%CI 776-5201 than those without 7737 95%CI 4907-10567 p=0.013. | N/A | N/A |
| Beach *et al.,* 2017 [22]. | ICU | **Steps per day.**  Median of 4[1.2-7] | **Minutes of PA per day:**  Median of 16.8 [0.6-152.4]  **Participants undertaking PA >100 mins of activity per day:**  1 (2%) participants on day 1 &  14 (25%) of participants on day 3. | N/A |
| Estrup *et al.,* 2018 [28]. | Ward following discharge from the ICU | **The median of the mean activity counts:**  104,634 (IQR 65,569 - 165,624)  **Mean activity during daytime:**  43,699 (Daytime 7am-4pm) | N/A | N/A |
| Schujmann *et al.,* 2019 [20]. | N/A | **Steps per day:**  591 ± 403 control, 1539 ± 966 intervention. (At ICU discharge) | **PA (% of the time):**  Light 3.85+1.9 control, 6.4+2.4 IG,  moderate 0.3+0.2 control, 1.012+0.6 IG,  Intense 0.03+0.02 control, 0.15+0.10 IG. | **(% of the time) inactive:** 95.7+2 control,  92.3+2.8 intervention. |
| Baldwin *et al*., 2020- [13]. | ICU, followed by hospital ward to discharge from hospital | N/A | **Number of activity bouts:**  At awakening:  ≥2 mins 2.9 (5.4)  ≥5 mins 1.1 (2.8),  At ICU discharge:  ≥2 mins 9.2 (12.7)  ≥ 5min 3.2 (4.8)  At hospital discharge:  ≥2 mins 17.7 (14.8)  ≥5 mins 5.8 (5.9) (SD in brackets)  **Time spent in activity (mins) (≥200g/min):**  At awakening: 27.0 (54.7)  At ICU discharge: 61.1 (98.8)  At Hospital discharge: 96.8 (85.1) (SD in brackets) | **% of time spent in SB:**  At awakening 98.1%  At ICU discharge 95.7%  At hospital discharge 93%  **No. of lying/sitting bouts:**  At awakening:  ≥30 min 2.5 (2.1),  ≥2 hrs 1.9 (1.4);  at ICU discharge:  ≥30 mins 5.1 (3.3),  ≥2hrs 3.0 (1.5);  at Hospital discharge:  ≥30 mins 9.3 (4.5)  ≥2hrs 3.4 (1.5).  **Number of upright bouts:**  At awakening:  ≥2 min 0.9 (2.4)  ≥5min 0.3 (1.0),  at ICU discharge:  ≥2 mins 2.3 (3.3)  ≥5min 0.6 (1.1),  at hospital discharge:  ≥2 min 7.2 (4.7)  ≥5min 2.9 (2.7) (SD in brackets) |
| Camus-Molina *et al.*, 2020 [36]. | ICU | **Activity counts:**  Awakening: 99,396 (31,707-193,692) ICU discharge: 309,104 (133,737-557,149)  **Activity counts per day:**  Awakening: 31,590 (21,275-52,219)  ICU discharge: 50,483 (38,073-88,649) | **Activity (minutes/day):**  Awakening: 17.8 (11.5-26.9)  ICU discharge: 35.5 (25.5-57.1) | **Inactivity time (min/day):** Awakening: 1422 (1413-1429)  ICU discharge: 1405 (1382.9-1414.5)  Total actigraphy recording time (median) from admission to ICU discharge 5.48 days (3.33-8.56)  0.15 days (0.06-0.25) equated to activity while 5.33 days (3.26-8.37) were inactive.  2.5% of time active, 97.5% of time inactive |
| Elias *et al.,* 2021 [37]. | Ward within 24 hours of ICU discharge | Post-ICU hourly activity averaged 2,233 ± 569 counts per hour | N/A | N/A |
| Elias *et al.,* 2021b [33]. | Ward within 24 hours of ICU discharge | The mean post-ICU 24-hour activity was 39.7+27.2 counts/min. | N/A | N/A |
| Elias *et al.,* 2021c [35]. | Ward within 24 hours of ICU discharge | Post-ICU daytime activity averaged 41.21 activity counts/min (sd28.24, range 5.8-91.2 [median=31.25, IQR 48.37] and nighttime activity averaged 29.27 activity counts/min (sd28.98, range 4 to 137.52, median 17.76; IQR 36.35) | N/A | N/A |
| Gandotra *et al*., 2021 [14]. | ICU, hospital ward, 7 days post-hospital discharge | **Daily step count (mean):**  Prior to ICU discharge: 95 [95% CI=15-173],  Prior to hospital discharge: 257 [95% CI= 114-400];  After hospital discharge: 1223 [95% CI= 376-2070]  4-6 days after discharge: 1278 [95% CI = 349-2207] | N/A | N/A |
| Munro *et al.,* 2021 [38]. | ICU | Most Hourly activity counts <1,000 | The mean DAR over the 5 days was 66.5 ± 19.2%; A DAR occurrence >80% was 17%. | N/A |
| Lehmkuhl *et al*., 2022 [32]. | ICU | N/A | **Mins spent moving:**  7; median, (3-11 IQR), 0-63 range.  **Mins spent in bed cycling:**  3; median, (0-20 IQR0, 0-125 range.  **Mins spent standing:**  1, median, (0-3 IQR0, 0-71 range;  **Mins spent walking:**  0 median, (0-0.02 IQR), 0-0.025 range.  1 hr standing, moving, walking, or bicycling while in ICU. | **Mins spent lying:**  1206 median, 917-1336 IQR, 199-1432 range;  **Mins spent sitting:**  179 median, 52-381 IQR, 0-1172 range.  MV patients spend 20/24hr lying in bed, 3hr sitting |
| Plekhanova *et al*., 2022 [25]. | 8 months post-hospital discharge | N/A | **Time spent in LIPA (mins/day):**  Women: 149.8 ± 54.9,  Men:138.8 ± 51.4 (mean ± SD).  **Time spent in MVPA (mins/day):**  Women:14.9 ± 14.8,  Men: 21.1 ± 22.3 (mean ± SD)  **Intensity of the most active (mg):**  Women: 10 min, 61.0 ± 39.0; 30 min, 40.4 ± 25.4;  Men: 10 min, 73.6 ± 64.0, 30 min, 49.6 ± 47.8  **Participants meeting >150 minutes of MVPA per week:**  Women: 68 (26.9%), Men: 172 (37.2%)  **No of days where a 10 moderate-intensity bout of PA was undertaken:**  0: Women, 139 (55.8%), Men, 199 (44.2%)  1: Women, 48 (19.3%), Men, 82 (18.2%)  2: Women, 28 (11.2%), Men, 58 (12.9%)  3+: Women, 34 (13.7%), Men, 111 (24.7%)  **No. of days where a 30 moderate-intensity bout of PA was undertaken:**  0: Women: 191 (76.7%), Men: 285 (63.3%)  1: Women; 33 (13.3%), Men; 68 (15.1%)  2: Women: 14 (5.6%), Men: 45 (10%)  3+: Women: 11 (4.4%), Men: 52 (11.6%) | **Mean Inactive hours/day:**  Women: 12.1 ± 1.7 (SD)  Men: 12.6 ± 1.7 , (SD)  **Mean Sleep hours/day:**  Women: 7.2 ±1.1 (SD)  Men: 6.9 ±1.1 (SD) |
| Rollinson *et al.,* 2022 [34]. | ICU to hospital ward | N/A | **Mean (sd) daily duration of PA >1.5METs**: ICU: 17.8(22.8) mins  Ward: 52.8(51.2) mins,  (mean difference (95% confidence interval) 35 (23.8-46.1) p<0.001) | N/A |
| Van Bakel *et al.,* 2022 [39]. | 3-6 months post-hospital discharge | N/A | **Time (hrs/day) spent in activity:**  Total cohort (n=37)  MVPA: 1.0 (0.8; 11.4). Median (IQR)  LIPA 4.2 (3.2; 5.3) Median (IQR)  ICU population N=13  LIPA: 4.2 (hrs) ± 1.4 Mean ± SD  MVPA: 1.2 (0.7; 1.3) Median (IQR) | **Sedentary time (hours/day):**  Total cohort (N=37):  9.8 (8.7; 11.2) Median (IQR)  ICU population N=13:  Hours sitting 9.9 ± 1.6 Mean ± SD |
| **Abbreviations: N/A: Not applicable, PA; Physical activity, SB: Sedentary behaviour, DAR: Daily activity ratio, MVPA: Moderate to vigorous physical activity, LIPA: Light intensity physical activity** | | | | |

### Step counts

Step counts were reported in 8/22 studies [14,20-24,26,31] Step counts were initially low but increased progressively from ICU to hospital discharge and home recovery. After awakening in ICU, ambulation was almost non-existent, with a median of four steps/day [22]. Daily step counts gradually increased to 95 at three days before ICU discharge, and 257 three days before hospital discharge [14]. There were minimal increases in daily step counts from 1,223 steps at three days and 1,278 steps up to eight days after hospital discharge [14]. At 18 months post-hospital discharge, daily step counts had risen to 5,803 steps, which was still significantly lower (p<0.001) than healthy age-matched controls (i.e. 11,735 steps) [23].

### Activity counts

Activity counts were reported in nine studies [27-30,33,35-38]. At awakening, a mean of 31,590 counts per day was reported, increasing to a mean of 50,483 counts per day at ICU discharge [36]. Activity counts averaged 2,233 per hour, within 24-48 hours of ICU discharge, where 1,140 counts per day equate to resting or lying down while awake [37]. Following discharge from the ICU, mean activity counts during the daytime (7am-4pm) were shown to be 43,699 [28].

Eight studies reported on the time spent being physically active [13,21,22,25,32,34,36,39]. During the ICU admission, time spent being physically active was low, with values of 17.8, 16.8 & 9.3 minutes/day [21,22,36]. This time increased at ICU discharge to 35.5 minutes per day [36], and to the hospital ward was 52.8 minutes per day [34]. A similar trend was shown by Baldwin [13] and colleagues, where time spent being physically active (>200g/min) increased from awakening (27 minutes) to 61 minutes at ICU discharge and to 96.8 minutes at hospital discharge [13].

### Sedentary behaviour

Sedentary behaviour was reported in eight studies [13,20,24,25,27,32,36,39]. These studies show the trajectory and change in sedentary behaviour over time, from awakening in the ICU [13,36] to eight months post-hospital discharge [25]. High levels of sedentary behaviour and inactivity were reported in the ICU. At awakening, Baldwin *et al*. [13] reported that their participants spent 98.1% of their time sedentary.

At ICU discharge, many participants typically spent 97.5% of their day being inactive [36]. Those in the study by Baldwin *et al.* [13] reduced their percentage of time spent inactive to 95.7% at ICU discharge, while this number reduced further to 93% at hospital discharge. At three months post-hospital discharge, sitting or lying time was reduced to 58% (from 89.2% in the hospital ward) by those observed by Borges *et al*. [24].

# Discussion

This scoping review has demonstrated that a wide range of devices have been used to measure physical activity and sedentary behaviour. There is a clear lack of standardisation regarding the data processing methods and there are no consistent parameters used when reporting physical activity levels or sedentary behaviour. There are some associations between physical activity and other outcomes such as physical function, strength, and exercise capacity. Overall, in people who have been admitted to ICU for critical illness, the levels of physical activity are generally low in ICU, on hospital wards and in the 18-month period after hospital discharge.

## Device-based instruments used to measure physical activity and sedentary behaviour

The most common devices used to measure physical activity and sedentary behaviour included the Actiwatch Spectrum, activPAL and SenseWear Armband. This finding reflects the wider literature related to accelerometry measurement in various clinical populations. However, researchers and clinicians should also be aware of the strengths and weaknesses of each of the devices. For example, a thigh-worn activPAL device may be more suited to tracking and recording sedentary behaviour and transitions from various positions, while potentially underestimating step counts [13]. If the goal is to measure positional changes, choosing this device or similar would be more appropriate. Future literature to support the use of a particular device and the protocols for wearing the device, including for example, location worn, should be based on research in the critical care population as opposed to healthy populations or other cohorts to be most applicable [40,41]. While various accelerometers have been utilised in individuals admitted to ICU, it is important for researchers and clinicians to be aware that there is likely to be a certain amount of measurement error with each device. For example, Baldwin and colleagues [26] compared activPAL-measured outcome variables with direct observation in ICU survivors. The authors found that although sit-to-stand transitions, sedentary behaviour and upright time were well-recognised by the activPAL, there were under-estimations of standing and stepping time compared to direct observation (22% and 19%, respectively). In healthy populations, free-living step counts have been shown to vary across seven activity monitors by around 1,700 steps/day (ranging from 1265 to 2275 steps/day) [42]. This shows that even though two activity monitors are supposedly measuring the same variable, there are likely to be error margins to consider. Unfortunately, few studies have currently explored validity and reliability of different devices in ICU survivors.

## Parameters of physical activity and sedentary behaviour

There are inconsistencies across the studies when defining physical activity and sedentary behaviour, the intensity of activity, and the thresholds used for the analysis of data on sedentary behaviour, making comparisons between studies difficult [43]. For the measurement of physical activity and sedentary behaviour, specific activity counts, milligravity (mg) units and METs have all been used. For METs, previous research has determined a cut-off point of <1.5 METs to define levels of sedentary behaviour, while 3 METs is commonly used as the cut-off point of LIPA/MVPA [44-46]. Survivors of ICU are often left with reduced exercise capacity and reduced levels of oxygen consumption, possibly due to mitochondrial dysfunction [47]. Therefore, it may be more appropriate to use lower MET values, such as those used in older populations, which could be as low as 2.8 ml/O2/kg [48-50] to avoid underestimation of the intensity of effort [51]. This was considered in one study [22], where a value of 1 MET was used (as opposed to 1.5 METs) as the threshold for physical activity. There are also other processing decisions that must be considered, such as epoch length, device placement and sampling frequency.

An epoch is defined as the time-period which allows data to be grouped together for analysis and usually varies from 1-60 seconds. Using different epoch lengths can affect the results by either over- or under-estimating physical activity and sedentary behaviour [52,53]. For example, the use of a 10-second epoch has been shown to increase the total daily sedentary behaviour time of older adults by almost 80 minutes when compared to a 60-second epoch [53]. The optimal epoch length for data analysis for assessing device-based physical activity within the critical care population has not been determined. In addition, the location the device is worn (e.g. thigh, wrist) alongside the dominant/non-dominant side worn have been shown to impact on important physical activity parameters, such as step counts. For example, daily steps have been shown to be significantly different between wrist and hip locations in adults wearing a pedometer [54], while another study [55] demonstrated dominant wrist placement resulted in 1,253 more daily steps compared to the non-dominant side (p=0.006).

The use of various sampling frequencies can also have an impact on the results and classification of physical activity and sedentary behaviour [56-58]. When using an ActiGraph GT3X, it has been recommended to use multiples of 30Hz [56,58], however, it is worth noting that using multiples of 30 may lead to filtering out of signals generated by vigorous activity and may result in an underestimation of vigorous activity [56].

The current heterogeneous approach to selecting and reporting parameters for the measurement of physical activity and sedentary behaviour is a clear limitation of the existing body of research as this impacts on the ability to compare and contrast study results or undertake meta-analysis. There is a need to clearly define and establish cut-points across the different MET thresholds, epoch lengths and sampling frequencies for analysis of physical activity data that relate to ICU survivors, as this would lead to a more consistent approach to measuring and reporting physical activity and sedentary behaviour. Table 3 provides a summary of some considerations and suggestions to help guide the use of device-based measurement in future critical care research and practice.

**Table 3. Practical considerations and suggestions to guide device based physical activity measurement and analysis for the critical care population.**

**Question or consideration and suggested guidance**

**Which device should I use?**

The most validated devices commonly used across various populations are ActiGraph [20,31,36], ActivPal [13,26,39] and Axivity [32].

**Where should I place the device?**

For optimal compliance: Wrist [58]

Optimal position for sedentary behaviour: thigh [58]

**Should I place on the dominant or non-dominant side?**

Depends on the device used. E.g. ActivPal non-dominant side [13,26,39] Main issue is to ensure consistent location is maintained.

**What should I provide to the patient/participant?**

Introduce the device to the participant, explaining what it does and does not do.

Provide a brief instruction manual, including information such as proper fitting of the device, whether to remove it for sleep, and information on items such as the meaning of different light alerts.

Recommend the participant fill in an activity monitor diary, to aid memory about when the device is worn and to record any error warning. This will also help understand when the device has been removed for data processing.

If meeting the participant after they have worn the device in free-living conditions, ask them to not take off the device, but wear it into the clinic/study visit location.

**How should I introduce the device to the participant to ensure optimal data collection?**

Follow the device manufacturer's guidelines for fitting the device. Also, refer to recommendations developed for other relevant clinical populations [59, 60].

Researchers should publish the procedure/protocol they used with the device in their study so that it is visible and will allow for replication of the study [61].

**How many days should the device be worn for?**

Optimal recommendation: 7 days [58]

Minimum for a valid data set:

-Post ICU: ≥2 days [24,27].

-Free-living: ≥4 days (1 Weekend) [58]

**What is considered a valid day?**

Regarding 24-hour protocols, there’s- currently no consensus, however, minimum values have included ≥10 hours [62,63] and ≥20 hours [9,64]

If using a waking hours-only protocol, the optimal/best practice should be ≥10 hours [53,59].

**What algorithms should be used to calculate non-wear time?**

This will depend on the selected device, the manufacturer's instructions, and the age of the cohort [58,65].

**What outcomes should I consider?**

Ideally, 24-hour health behaviours should be considered (i.e. physical activity, sleep, and sedentary behaviour).

**How should sedentary behaviour be expressed?**

The daily duration (time) and expressed as a percentage of wear time alongside daily time spent in prolonged bouts (e.g. 30 minutes) [13,24,39].

**How should physical activity be expressed?**

Overall summaries (e,g, step counts, vector magnitude units), daily durations, expressed as a percentage of wear-time, in light and moderate-vigorous physical activity, as well as considering the number and time spent in different bout lengths (e.g. 10 minutes) [13,20].

**What thresholds should I use for determining sedentary behaviour and intensities in different physical activities?**

This depends on several factors, such as the age profile of the population as well as the device used and the device placement [51, 58,65].

To overcome the challenges of measuring step counts in those with slow gait speed, and misclassification with standing, a low-frequency filter may be used (LFE). However, it is important to note that the use of a LFE may end up being overly sensitive. Some studies have demonstrated inflated step counts in adults when using the LFE [53,66,67], in some instances by up to 5,502 steps per day [53,66]. It is worth noting that Anderson *et al.* [31] used a LFE filter and concluded it provided a valid measurement of step counts from an ankle worn ActiGraph in hospital inpatients recovering from critical illness. In line with the recommendations for the COPD population, validation of devices to accurately measure step count using the LFE should be determined before use in the critical illness population [67].

Decisions on minimum daily wear time also need to be carefully considered, to appropriately represent activity and sedentary data while also maintaining sample sizes [53,58]. A valid daily wear time of ≥600 minutes (Table 3) has been suggested [53], and the results of this review show that only four studies met this criterion [13,23,25,32].

## Clinimetric properties

An understanding of the clinimetric properties of devices used in the ICU population is important to help guide the selection of the appropriate device for research. While studies have shown promise in validating devices for the critical care population when used under controlled conditions [13,31], researchers also cite validation studies from other populations (COPD, older adults) and this might not be the best approach. No studies in this review reported an MCID for physical activity or sedentary behaviour variables in this population, making it difficult to establish the change that is important to the patient or to establish the clinical effectiveness of an intervention [68,69]. This gap needs to be addressed in future research.

## Relationship between physical activity and sedentary behaviour with other health-related outcomes

In this review, levels of physical activity were shown to correlate with physical function, which provides evidence of the validity of physical activity as a construct [13,14,20-23,28,33]. However, correlations between physical activity and other outcome measures such as cognitive function and frailty, that are recommended in core outcome sets, have not been extensively studied [70]. It is worth noting that not all studies showed significant correlations of physical activity with all the same health-related outcome measures. Potential reasons for this could be down to the specific device which was used in each study, but also the test used to collect the health-related outcome measure. For example, physical function was measured using a battery of four tests by Rollinson *et al.* [34], while Estrup *et al.* [28] used the Chelsea Critical Care Physical Assessment tool (CPAx) which assesses ten aspects of physical function. These two studies had contrasting findings when it came to the association of physical function with physical activity.

## Levels of physical activity and sedentary behaviour

Physical activity levels in ICU survivors are low compared to healthy populations, even up to 18 months post-discharge [23], with a high prevalence of sedentary behaviour. This could contribute to the negative long-term effects of sedentary behaviour that have been well-documented [8]. While it is important to increase levels of physical activity in ICU survivors [11], efforts should also be made to encourage less sedentary behaviour where possible [71,72]. Prolonged periods of sedentary behaviour when recumbent (lying) may result in little-to-no activity counts being recorded on the device and may give the impression of sleep. Similar challenges were found in the COPD population, and the algorithm developed for using the ActiGraph wGT3X-BT accelerometer to help determine when the person may be sleeping or indeed waking up could be useful [62].

Only one of the 22 included studies was a randomised control trial investigating the effect of an intervention (a mobility programme) on physical activity in a cohort of patients within 48 hours of admission to ICU [18]. Improvements reported in the study were consistent with findings supporting the benefits of early mobilisation in the ICU [73], therefore, accelerometry could be an applicable outcome in future research exploring effects of early mobilisation in the ICU or investigating the effectiveness of rehabilitation interventions after ICU discharge. High-quality experimental studies are required to establish cause and effect relationships between physical activity and sedentary behaviour with important patient outcomes. At present, nearly all research is observational in nature. Therefore, there is a clear need for future experimental studies exploring the impact of changing physical activity and sedentary behaviour on important patient outcomes. Investigation is also needed to test the best way to support recovery of physical activity and reduction of excessive sedentary behaviour following discharge from hospital.

The strengths of this scoping review include a comprehensive search strategy and pre-identified rigorous methodology (15,16) as well as the inclusion of studies across the entire recovery trajectory following ICU. The review protocol was registered *a priori* to ensure transparency. Limitations of this scoping review were that we did not contact authors to ask whether further details about their data processing could be made available and our results were limited by the lack of a standardised approach to measurement used in the included papers making it difficult to compare across studies.

In future, researchers and clinicians will need to be able to select appropriate device-based physical activity instruments to accurately assess physical activity and sedentary behaviour. This review highlights the devices that could be used and the associated challenges. There are some existing guidelines for the assessment of physical activity in other populations [59,60]. There is a clear need for an agreed protocol(s) to guide the processing and analysis of data for the critical care population, therefore, to address this limitation, we have made some practical suggestions/recommendations that researchers and clinicians could use until such evidence-informed guidelines are available (Table 3). Future research may also benefit from the use of machine learning to develop protocols and assist in differentiating between different physical activity behaviours/patterns [74].

# Conclusion

The use of device-based physical activity measurement in critical illness is an active and evolving research area. A serious issue we have highlighted is that there appears to be inconsistent reporting and usage of various physical activity and sedentary behaviour parameters related to accelerometry in critical illness. This review supports the need for the development of a strategy or protocol to guide the future use of devices and standardise processing and analysis when assessing physical activity and sedentary behaviour in the population with critical illness. In addition, there is a need for researchers to establish the typical MET values required for completing common types of physical activity specifically in this population, in order to guide more effective physical activity prescription. Physical activity levels remain low in both ICU and following ICU discharge, and investment is needed to support recovery of physical activity and reduce sedentary behaviour following discharge from hospital.

## Acknowledgements

The authors would like to acknowledge Kelly Coogan, assistant subject librarian, faculty of life & health science, Ulster University, in providing expertise and assisting with developing and conducting the search strategy.

# References

1. Pfoh ER, Wozniak AW, Colantuoni E, Dinglas VD, Mendez-Tellez PA, Shanholtz C, et al. Physical declines occurring after hospital discharge in ARDS survivors: a 5-year longitudinal study. Intensive Care Med. 2016;42(10):1557–66.doi:10.1007/s00134-016-4530-1
2. Hopkins RO, Suchyta MR, Kamdar BB, Darowski E, Jackson JC, Needham DM. Instrumental Activities of Daily Living after Critical Illness: A Systematic Review. Ann Am Thorac Soc. 2017; 14(8):1332–43. doi:10.1513/annalsats.201701-059sr
3. Rai R, Singh R, Azim A, Agarwal A, Mishra P, Singh PK. Impact of Critical Illness on Quality of Life after Intensive Care Unit Discharge. Indian J Crit Care Med. 2020;24(5):299–306. doi:10.5005/jp-journals-10071-23433
4. Nedergaard HK, Haberlandt T, Reichmann PD, Toft P, Jensen HI. Patients' opinions on outcomes following critical illness. Acta Anaesthesiol Scand. 2018;62(4):531–9. doi:10.1111/aas.13058
5. Bull FC, Al-Ansari SS, Biddle S, Borodulin K, Buman MP, Cardon G, et al. World Health Organization 2020 guidelines on physical activity and sedentary behaviour. Br J Sports Med. 2020;54(24):1451–62. doi:10.1136/bjsports-2020-102955
6. de Rezende LFM, Rey-López JP, Matsudo VKR, do Carmo Luiz O. Sedentary behavior and health outcomes among older adults: a systematic review. BMC Public Health. 2014;14:333. doi:10.1186/1471-2458-14-333
7. de Rezende LFM, Rodrigues Lopes M, Rey-López JP, Matsudo VKR, Luiz OdC. Sedentary behavior and health outcomes: an overview of systematic reviews. PLoS One. 2014;9(8):e105620. doi:10.1371/journal.pone.0105620
8. Pinto AJ, Bergouignan A, Dempsey PC, Roschel H, Owen N, Gualano B, et al. Physiology of sedentary behavior. Physiol Rev. 2023;103(4):2561–622. doi:10.1152/physrev.00022.2022
9. Blodgett JM, Ahmadi MN, Atkin AJ, Chastin S, Chan H, Suorsa K, et al. Device-measured physical activity and cardiometabolic health: the Prospective Physical Activity, Sitting, and Sleep (ProPASS) consortium. Eur Heart J. 2024;45(6):458–71. doi:10.1093/eurheartj/ehad717
10. Angelucci A, Greco M, Cecconi M, Aliverti A. Wearable devices for patient monitoring in the intensive care unit. Intensive Care Med Exp 2025;13(1):26. doi:10.1186/s40635-025-00738-8
11. Eggmann S, Timenetsky KT, Hodgson C. Promoting optimal physical rehabilitation in ICU. Intensive Care Med. 2024;50(5):755–7. doi:10.1007/s00134-024-07384-w
12. Gluck S, Chapple LS, Chapman MJ, Iwashyna TJ, Deane AM. A scoping review of use of wearable devices to evaluate outcomes in survivors of critical illness. Crit Care Resusc. 2017;19(3):197–204.
13. Baldwin CE, Rowlands AV, Fraysse F, Johnston KN. The sedentary behaviour and physical activity patterns of survivors of a critical illness over their acute hospitalisation: An observational study. Aust Crit Care. 2020;33(3):272–80. doi:10.1016/j.aucc.2019.10.006
14. Gandotra S, Files DC, Shields KL, Berry M, Bakhru RN. Activity Levels in Survivors of the Intensive Care Unit. Phys Ther. 2021;101(9):pzab135. doi:10.1093/ptj/pzab135
15. Munn Z, Peters MDJ, Stern C, Tufanaru C, McArthur A, Aromataris E. Systematic review or scoping review? Guidance for authors when choosing between a systematic or scoping review approach. BMC Med Res Methodol 2018;18(1):143. doi: 10.1186/s12874-018-0611-x
16. Arksey H, O'malley L. Scoping studies: towards a methodological framework. International journal of social research methodology. 2005;8(1):19–32. doi: 10.1080/1364557032000119616
17. Levac D, Colquhoun H, O'brien KK. Scoping studies: advancing the methodology. Implementation science. 2010;5:1–9. doi:10.1186/1748-5908-5-69
18. Tricco AC, Lillie E, Zarin W, O'Brien KK, Colquhoun H, Levac D, et al. PRISMA Extension for Scoping Reviews (PRISMA-ScR): Checklist and Explanation. Ann Intern Med 2018;169(7):467–73. doi:10.7326/M18-0850
19. Haddaway NR, Page MJ, Pritchard CC, McGuinness LA. PRISMA2020: An R package and Shiny app for producing PRISMA 2020-compliant flow diagrams, with interactivity for optimised digital transparency and Open Synthesis. Campbell Syst Rev 2022;18(2):e1230. doi:10.1002/cl2.1230
20. Schujmann DS, Teixeira Gomes T, Lunardi AC, Zoccoler Lamano M, Fragoso A, Pimentel M, et al. Impact of a Progressive Mobility Program on the Functional Status, Respiratory, and Muscular Systems of ICU Patients: A Randomized and Controlled Trial. Crit Care Med 2020;48(4):491–7. doi:10.1097/CCM.0000000000004181
21. Beach LJ, Fetterplace K, Edbrooke L, Parry SM, Curtis R, Rechnitzer T, et al. Low physical activity levels and poorer muscle strength are associated with reduced physical function at intensive care unit discharge: an observational study. C44. CRITICAL ILLNESS PREDICTORS AND OUTCOMES: American Thoracic Society; 2014. p. A4543.
22. Beach LJ, Fetterplace K, Edbrooke L, Parry SM, Curtis R, Rechnitzer T, et al. Measurement of physical activity levels in the Intensive Care Unit and functional outcomes: An observational study. J Crit Care. 2017;40:189–96. doi:10.1016/j.jcrc.2017.04.006
23. McNelly AS, Rawal J, Shrikrishna D, Hopkinson NS, Moxham J, Harridge SD, et al. An Exploratory Study of Long-Term Outcome Measures in Critical Illness Survivors: Construct Validity of Physical Activity, Frailty, and Health-Related Quality of Life Measures. Crit Care Med. 2016;44(6):362. doi:10.1097/CCM.0000000000001645
24. Borges RC, Carvalho CRF, Colombo AS, da Silva Borges MP, Soriano FG. Physical activity, muscle strength, and exercise capacity 3 months after severe sepsis and septic shock. Intensive Care Med. 2015;41(8):1433–44. doi:10.1007/s00134-015-3914-y
25. Plekhanova T, Rowlands AV, Evans RA, Edwardson CL, Bishop NC, Bolton CE, et al. Device-assessed sleep and physical activity in individuals recovering from a hospital admission for COVID-19: a multicentre study. Int J Behav Nutr Phys Act. 2022;19(1):94. doi:10.1186/s12966-022-01333-w
26. Baldwin CE, Johnston KN, Rowlands AV, Williams MT. Physical Activity of ICU Survivors during Acute Admission: Agreement of the activPAL with Observation. Physiother Can 2018;70(1):57–63. doi:10.3138/ptc.2016-61
27. Gupta P, Martin JL, Needham DM, Vangala S, Colantuoni E, Kamdar BB. Use of actigraphy to characterize inactivity and activity in patients in a medical ICU. Heart Lung. 2020;49(4):398–406. doi:10.1016/j.hrtlng.2020.02.002
28. Estrup S, Kjer CKW, Vilhelmsen F, Poulsen LM, Gøgenur I, Mathiesen O. Physical function and actigraphy in intensive care survivors-A prospective 3-month follow-up cohort study. Acta Anaesthesiol Scand. 2019 -05;63(5):647–52. doi:10.1111/aas.13317
29. Winkelman C. Investigating activity in hospitalized patients with chronic obstructive pulmonary disease: a pilot study. Heart Lung. 2010;39(4):319–30. doi:10.1016/j.hrtlng.2009.09.004
30. Grap MJ, Borchers CT, Munro CL, Elswick RK, Sessler CN. Actigraphy in the critically ill: correlation with activity, agitation, and sedation. Am J Crit Care. 2005;14(1):52–60.
31. Anderson JL, Yoward LS, Green AJ. A study investigating the validity of an accelerometer in quantification of step count in adult hospital inpatients recovering from critical illness. Clin Rehabil. 2019;33(5):936–42. doi:10.1177/0269215519829893
32. Lehmkuhl L, Olsen HT, Brønd JC, Rothmann MJ, Dreyer P, Jespersen E. Daily variation in physical activity during mechanical ventilation and stay in the intensive care unit. Acta Anaesthesiol Scand. 2023;67(4):462–9. doi:10.1111/aas.14195
33. Elías MN, Munro CL, Liang Z. Daytime activity and sleep are associated with motor function in older intensive care unit survivors. Heart Lung. 2021;50(4):542–5. doi:10.1016/j.hrtlng.2021.02.008
34. Rollinson TC, Connolly B, Berlowitz DJ, Berney S. Physical activity of patients with critical illness undergoing rehabilitation in intensive care and on the acute ward: An observational cohort study. Aust Crit Care. 2022;35(4):362–8. doi:10.1016/j.aucc.2021.06.005 PMID: 34389239
35. Elías MN, Munro CL, Liang Z. Daytime Activity Is Associated With Discharge to Home in Older Adults Recovering From Critical Illness. J Gerontol Nurs. 2021;47(2):13–19. doi:10.3928/00989134-20210107-03
36. Camus-Molina A, González-Seguel F, Castro-Ávila AC, Leppe J. Construct Validity of the Chilean-Spanish Version of the Functional Status Score for the Intensive Care Unit: A Prospective Observational Study Using Actigraphy in Mechanically Ventilated Patients. Arch Phys Med Rehabil. 2020;101(11):1914–21. doi:10.1016/j.apmr.2020.04.019
37. Elías MN, Munro CL, Liang Z. Graphic Representation of Hourly Activity Counts May Identify Discharge Outcomes for Older Adults After Critical Illness. Rehabil Nurs. 2021;46(5):247–52. doi:10.1097/rnj.0000000000000312
38. Munro CL, Liang Z, Elías MN, Ji M, Chen X, Calero K. Sleep and Activity Patterns Are Altered During Early Critical Illness in Mechanically Ventilated Adults. Dimens Crit Care Nurs. 2021;40(1):29–35. doi:10.1097/DCC.0000000000000455
39. van Bakel BMA, van den Heuvel FMA, Vos JL, Rotbi H, Bakker EA, Nijveldt R, et al. High Levels of Sedentary Time in Patients with COVID-19 after Hospitalisation. J Clin Med. 2022;11(4):1110. doi:10.3390/jcm11041110
40. Troiano RP, McClain JJ, Brychta RJ, Chen KY. Evolution of accelerometer methods for physical activity research. Br J Sports Med. 2014; 48(13):1019–23. doi:10.1136/bjsports-2014-093546
41. Wolpern AE, Sherwin KJ, Moss WD, Nygaard IE, Egger MJ, Brusseau TA, et al. Compliance with wrist-worn accelerometers in primiparous early postpartum women. Heliyon. 2019;5(1):e01193. doi:10.1016/j.heliyon.2019.e01193
42. Nakagata T, Yamada Y, Taniguchi M, Nanri H, Kimura M, Miyachi M, et al. Comparison of step-count outcomes across seven different activity trackers: a free-living experiment with young and older adults. BMC Sports Sci Med Rehabil 2024;16(1):156. doi:10.1186/s13102-024-00943-0
43. Kim Y, Barry VW, Kang M. Validation of the ActiGraph GT3X and activPAL accelerometers for the assessment of sedentary behavior. Measurement in Physical Education and Exercise Science. 2015;19(3):125–37. doi:10.1080/1091367X.2015.1054390
44. Ainsworth BE, Haskell WL, Herrmann SD, Meckes N, Bassett Jr DR, Tudor-Locke C, et al. 2011 Compendium of Physical Activities: a second update of codes and MET values. Medicine & science in sports & exercise. 2011;43(8):1575–81. doi:10.1249/MSS.0b013e31821ece12
45. Herrmann SD, Willis EA, Ainsworth BE, Barreira TV, Hastert M, Kracht CL, et al. 2024 Adult Compendium of Physical Activities: A third update of the energy costs of human activities. J Sport Health Sci. 2024;13(1):6–12. doi:10.1016/j.jshs.2023.10.010
46. Tremblay MS, Aubert S, Barnes JD, Saunders TJ, Carson V, Latimer-Cheung AE, et al. Sedentary Behavior Research Network (SBRN) - Terminology Consensus Project process and outcome. Int J Behav Nutr Phys Act. 2017;14(1):75. doi:10.1186/s12966-017-0525-8
47. Mart MF, Ely EW, Tolle JJ, Patel MB, Brummel NE. Physiologic responses to exercise in survivors of critical illness: an exploratory pilot study. Intensive Care Med Exp. 2022;10(1):35. doi: 10.1186/s40635-022-00461-8
48. Leal-Martín J, Muñoz-Muñoz M, Keadle SK, Amaro-Gahete F, Alegre LM, Mañas A, et al. Resting Oxygen Uptake Value of 1 Metabolic Equivalent of Task in Older Adults: A Systematic Review and Descriptive Analysis. Sports Med. 2022;52(2):331–48. doi:10.1007/s40279-021-01539-1
49. Savage PD, Toth MJ, Ades PA. A re-examination of the metabolic equivalent concept in individuals with coronary heart disease. J Cardiopulm Rehabil Prev. 2007;27(3):143–8. doi:10.1097/01.HCR.0000270693.16882.d9
50. Skjødt M, Tully MA, Tsai L, Gejl KD, Ørtenblad N, Jensen K, et al. Need to revise classification of physical activity intensity in older adults? The use of estimated METs, measured METs and V̇O2Reserve. J Gerontol A Biol Sci Med Sci. 2024;79(8):glae120. doi:10.1093/gerona/glae120
51. Barnett A, van den Hoek D, Barnett D, Cerin E. Measuring moderate-intensity walking in older adults using the ActiGraph accelerometer. BMC Geriatr. 2016;16:211. doi:10.1186/s12877-016-0380-5
52. Clevenger KA, Brønd JC, Mackintosh KA, Pfeiffer KA, Montoye AHK, McNarry MA. Impact of ActiGraph sampling rate on free-living physical activity measurement in youth. Physiol Meas. 2022;43(10):105004. doi:10.1088/1361-6579/ac944f
53. Wilson JJ, Skjødt M, McMullan I, Blackburn NE, Giné-Garriga M, Sansano-Nadal O, et al. Consequences of choosing different settings when processing hip-based accelerometry data from older adults: a practical approach using baseline data from the SITLESS study. Journal for the Measurement of Physical Behaviour. 2020;3(2):89–99. doi:10.1123/jmpb.2019-0037
54. Park S, Toth LP, Crouter SE, Springer CM, Marcotte RT, Bassett DR. Effect of monitor placement on the daily step counts of wrist and hip activity monitors. Journal for the Measurement of Physical Behaviour 2020;3(2):164–169. doi:10.1123/jmpb.2019-0065
55. Park S, Toth LP, Hibbing PR, Springer CM, Kaplan AS, Feyerabend MD, et al. Dominant vs. non-dominant wrist placement of activity monitors: impact on steps per day. Journal for the Measurement of Physical Behaviour 2019;2(2):118–123. doi:10.1123/jmpb.2018-0060
56. Brønd JC, Arvidsson D. Sampling frequency affects the processing of Actigraph raw acceleration data to activity counts. J Appl Physiol. (1985) 2016;120(3):362–9. doi:10.1152/japplphysiol.00628.2015
57. Clevenger KA, Brønd JC, Arvidsson D, Montoye AH, Mackintosh KA, McNarry MA, et al. Impact of ActiGraph sampling rate and intermonitor comparability on measures of physical activity in adults. Journal for the Measurement of Physical Behaviour. 2021;4(4):287–97. doi:10.1123/jmpb.2021-0016
58. Migueles JH, Cadenas-Sanchez C, Ekelund U, Delisle Nyström C, Mora-Gonzalez J, Löf M, et al. Accelerometer Data Collection and Processing Criteria to Assess Physical Activity and Other Outcomes: A Systematic Review and Practical Considerations. Sports Med 2017;47(9):1821–45. doi:10.1007/s40279-017-0716-0
59. Vetrovsky T, Clark CCT, Bisi MC, Siranec M, Linhart A, Tufano JJ, et al. Advances in accelerometry for cardiovascular patients: a systematic review with practical recommendations. ESC Heart Fail. 2020;7(5):2021–31. doi:10.1002/ehf2.12781
60. Dall PM, Skelton DA, Dontje ML, Coulter EH, Stewart S, Cox SR, et al. Characteristics of a protocol to collect objective physical activity/sedentary behaviour data in a large study: Seniors USP (understanding sedentary patterns). Journal for the measurement of physical behaviour. 2018;1(1):26-31. doi:10.1123/jmpb.2017-0004
61. Hoffmann TC, Glasziou PP, Boutron I, Milne R, Perera R, Moher D, et al. Better reporting of interventions: template for intervention description and replication (TIDieR) checklist and guide. BMJ. 2014;348 doi:10.1136/bmj.g1687
62. Orme MW, Steiner MC, Morgan MD, Kingsnorth AP, Esliger DW, Singh SJ, et al. 24-hour accelerometry in COPD: Exploring physical activity, sedentary behavior, sleep and clinical characteristics. Int J Chron Obstruct Pulmon Dis. 2019;14:419–30. doi:10.2147/COPD.S183029
63. Wennman H, Pietilä A, Rissanen H, Valkeinen H, Partonen T, Mäki-Opas T, et al. Gender, age and socioeconomic variation in 24-hour physical activity by wrist-worn accelerometers: the FinHealth 2017 Survey. Sci Rep. 2019;9(1):6534 doi:10.1038/s41598-019-43007-x
64. Blodgett JM, Bann D, Chastin SFM, Ahmadi M, Stamatakis E, Cooper R, et al. Socioeconomic gradients in 24-hour movement patterns across weekends and weekdays in a working-age sample: evidence from the 1970 British Cohort Study. J Epidemiol Community Health. 2024;78(8):515–21. doi:10.1136/jech-2023-221726
65. Published cut-points and how to use them in GGIR [Internet]. R-project.org. 2015 [cited 2024 Oct 24]. Available from: https://cran.r-project.org/web/packages/GGIR/vignettes/CutPoints.html#1_Consideration
66. Feito Y, Hornbuckle LM, Reid LA, Crouter SE. Effect of ActiGraph’s low frequency extension for estimating steps and physical activity intensity. PLoS One. 2017;12(11):e0188242. doi:10.1371/journal.pone.0188242
67. Antão J, Rebelo P, Almeida S, Franssen FME, Spruit MA, Marques A. Effects of ActiGraph's filter, epoch length and non-wearing time algorithm on step counts in people with COPD. J Sports Sci. 2024;42(1):9–16. doi:10.1080/02640414.2024.2319448
68. Demeyer H, Burtin C, Hornikx M, Camillo CA, Van Remoortel H, Langer D, et al. The Minimal Important Difference in Physical Activity in Patients with COPD. PLoS One. 2016;11(4):e0154587. doi:10.1371/journal.pone.0154587
69. Gardner AW, Montgomery PS, Wang M, Shen B. Minimal clinically important differences in daily physical activity outcomes following supervised and home-based exercise in peripheral artery disease. Vasc Med. 2022;27(2):142–9. doi:10.1177/1358863X211072913
70. Connolly BA, Barclay M, Davies C, Hart N, Pattison N, Sturmey G, et al. PRACTICE: Development of a Core Outcome Set for Trials of Physical Rehabilitation in Critical Illness. Ann Am Thorac Soc. 2024;21(12):1742–50. doi:10.1513/AnnalsATS.202406-581OC
71. Chrisman M, Chesnut SR, Thompson M, Hopper A, Lasiter S. Physical activity and sedentary behavior in middle-aged intensive care unit survivors discharged home: A systematic review. Intensive Crit Care Nurs. 2024;81:103608. doi:10.1016/j.iccn.2023.103608
72. Mavroeidi A, McInally L, Tomasella F, Dall PM, Skelton DA. An explorative study of current strategies to reduce sedentary behaviour in hospital wards. AIMS Medical Science. 2019;6(4):285–95. doi:10.3934/medsci.2019.4.285
73. Zhang L, Hu W, Cai Z, Liu J, Wu J, Deng Y, et al. Early mobilization of critically ill patients in the intensive care unit: A systematic review and meta-analysis. PLoS One. 2019;14(10):e022318 doi:10.1371/journal.pone.0223185
74. Farrahi V, Rostami M. Machine learning in physical activity, sedentary, and sleep behavior research. Journal of Activity, Sedentary and Sleep Behaviors. 2024; 3(1):5. doi:10.1186/s44167-024-00045-9

## Supporting information

**S1. Search strategy**

**S2 Table. Study characteristics**

**S3 Table. Correlations device measured physical activity and other health outcomes measured**

**S4. PRISMA-ScR Checklist**
